# High-throughput imaging of powdery mildew resistance of the winter wheat collection hosted at the German Federal ex situ Genebank for Agricultural and Horticultural Crops

**DOI:** 10.1093/gigascience/giad007

**Published:** 2023-03-03

**Authors:** Valentin Hinterberger, Dimitar Douchkov, Stefanie Lueck, Jochen C Reif, Albert W Schulthess

**Affiliations:** Leibniz Institute of Plant Genetics and Crop Plant Research (IPK), Department of Breeding Research, D-06466, Seeland, Germany; Leibniz Institute of Plant Genetics and Crop Plant Research (IPK), Department of Breeding Research, D-06466, Seeland, Germany; Leibniz Institute of Plant Genetics and Crop Plant Research (IPK), Department of Breeding Research, D-06466, Seeland, Germany; Leibniz Institute of Plant Genetics and Crop Plant Research (IPK), Department of Breeding Research, D-06466, Seeland, Germany; Leibniz Institute of Plant Genetics and Crop Plant Research (IPK), Department of Breeding Research, D-06466, Seeland, Germany

## Abstract

**Background:**

Genebanks worldwide are transforming into biodigital resource centers, providing access not only to the plant material itself but also to its phenotypic and genotypic information. Adding information for relevant traits will help boost plant genetic resources' usage in breeding and research. Resistance traits are vital for adapting our agricultural systems to future challenges.

**Findings:**

Here we provide phenotypic data for the resistance against *Blumeria graminis* f. sp. *tritici*, the causal agent of wheat powdery mildew—a substantial risk to our agricultural production. Using a modern high-throughput phenotyping system, we infected and photographed a total of 113,638 wheat leaves of 7,320 winter wheat (*Triticum aestivum* L.) plant genetic resources of the German Federal ex situ Genebank for Agricultural and Horticultural Crops and 154 commercial genotypes. We quantified the resistance reaction captured by images and provide them here, along with the raw images.

**Conclusion:**

This massive amount of phenotypic data, combined with already published genotypic data, also provides a valuable and unique training dataset for the development of novel genotype-based predictions as well as mapping methods.

## Background

Our agricultural system is facing one of the most significant upheavals in decades. In addition to uncertainties arising from ongoing climatic change and the ever-increasing demand for agricultural goods, the ecological impact of agricultural production is more than ever in the spotlight. In this context, the European “Farm to Fork Strategy” has set ambitious goals for a more sustainable agricultural production. One of these goals is to reduce pesticide use by 50% by 2030 [1]. Fungicides form an important group of pesticides in cereal crops, which have been used regularly in intensive agriculture since the mid-1970s. The reasons why there is an urgent need to reduce the use of fungicides are manifold: harmful pesticide residues [2], decreasing efficacy of active components due to pathogenic resistance [3], and side effects on the environment and the crops [4, 5] are just some of them.

There are many agronomical ways to reduce fungicide usage—for example, precision farming [6], improved crop rotation, changes in sowing date, and straw management. Growing resistant varieties is one of the easiest and most sustainable solutions for the farmer. While easy to adopt for the farmer, breeding a stable, resistant variety with excellent quality and high yield is a great challenge for breeders and phytopathologists. The past decades have shown continuous cycles of a “boom-and-bust” pattern in resistance development—new major qualitative resistance mechanisms are identified and heavily used in agriculture. This has led to a strong selection pressure on the pathogen population and an inevitable breakdown of the resistance by population shift and mutations [7, 8]. Especially biotrophic pathogens like *Blumeria graminis*, the causal agent of powdery mildew (PM), show a rapid and strong response to deploying new resistance mechanisms [8]. In this context, the risk of pathogen populations adapting to resistance mechanisms can be delayed by increasing diversity of the resistance mechanisms in cultivars and relying on quantitative resistance provided by the additive effect of several minor resistance genes [3, 7].

Providing donors for new, unused, or since a long-time abandoned resistance genes is one of the main purposes of genebanks like the German Federal ex situ Genebank for Agricultural and Horticultural Crops. The great challenge for breeders and scientists lies here in finding useful plant genetic resources (PGRs) among thousands of genebank accessions. In order to make these informed prebreeding decisions possible, we have tested almost all of the winter wheat (*Triticum aestivum* L.) collection at the Leibniz Institute of Plant Genetics and Crop Plant Research (IPK) for its quantitative resistance to PM by combining high-throughput imaging of detached leaf assays and a machine-based quantification of the percentage of infected leaf area. In this process, we infected and photographed a total of 113,638 wheat leaves of 7,320 accessions and 154 varieties used by farmers in Germany during the past decades. These data were obtained in a controlled environment at the seedling stage and using the highly virulent PM isolate FAL 92315. Under this highly controlled setup and provided a strong genotypic effect of host plants, fungal growth could be attributed to a quantitative resistance response of genotypes. Such a reliable association would most likely not be possible based on field data that rely on natural infections and much less controlled environmental conditions.

Detached leaf assays are a standard method in phytopathology to assess plant resistance in a cheap, fast, easy, and repeatable manner [9]. They are traditionally performed to measure the qualitative resistance response at the seedling stage of plants. However, there is evidence for quantitative resistance mechanisms in seedlings. For example, *Lr34* confers partial resistance already at the seedling stage [10], while some *SWEET* genes have been associated with quantitative susceptibility in seedlings [11, 12]. Some of those quantitative or partial resistance mechanisms have a delaying (latency) effect on the development of the pathogen, resulting in longer reproduction cycles and a reduced spore production by the pathogen [13]. We therefore investigated the plausibility of capturing latency mechanisms of quantitative resistance against PM at the seedling stage in a detached leaf assay setup applied at a large scale to genebank material.

The data presented here can be further extended with additional untested plant material by using the same environmental parameters and isolate. In addition, this dataset may help to develop or train new image analysis tools for images derived from detached leaf assays. In combination with additional analysis using other isolates of *B. graminis*, it can be part of a genotype-by-genotype analysis elucidating host–pathogen interactions. As a component of genome-wide mapping approaches, these data are a valuable source of information on donors for potentially novel resistance genes, as we recently have shown [14]. We expect that our quantitative resistance data contribute to the discovery of basal resistance mechanisms that provide a more durable crop protection in the future.

## Methods

### Plant material

The German Federal ex situ Genebank for Agricultural and Horticultural Crops, located at the IPK, hosts more than 27,000 wheat PGR of the *Triticum*sp. genus [15]. In this study, we present phenotypic data for PM resistance of 7,320 wheat PGR and 154 winter wheat varieties representing the cultivated varieties in Germany in the past decade (in the following denoted as the Elite Panel). In addition, a set of 929 additional genotypes (coded as Div_Set_1–929) were also tested in experiments but were not part of the study. Phenotypes of these additional genotypes were kept in the dataset to not disrupt the data structure and to allow proper correction for experimental design effects.

During field multiplication of genebank material, we used a “single-seed descent” (SSD) step to obtain defined seeds—for details, see [16, 17]. This was achieved by bagging 1 representative ear for each of 7,242 homogeneous accessions and 2 ears in case of 78 accessions, which we identified as clearly heterogeneous based on the morphological appearance of plants within each accession. For the genotypes of the Elite Panel, defined seeds were obtained from local seed market providers.

### High-throughput phenotyping of plant–pathogen interactions

The phenotypic data presented here were gathered using the Macrobot facility, a robotic platform performing high-throughput semiautomatic detached leaf assays [18, 19]. For the Macrobot assay, seedlings from defined seeds were grown in trays with 6 × 4 slots in the greenhouse under standardized conditions. In each slot, 10 seedlings of the same genotype were grown. For the inoculation assay, a leaf segment was cut from the second leaf of the 14-day-old seedlings. We cut the middle part of the leaf because early trials evidenced that the base of the leaf is more susceptible to powdery mildew, while the tip is more resistant (data not shown). The 2-cm-long leaf segments were brought onto microtiter agar plates. Each plate consisted of 4 lanes, each with leaf segments from up to 8 leaves per tested genotype. These plates were then infected with highly virulent *B. graminis*f. sp.*tritici* isolate FAL 92315 in a rotating platform by blowing spores from heavily infected leaves using a compressed air pistol.

**Figure 1:**
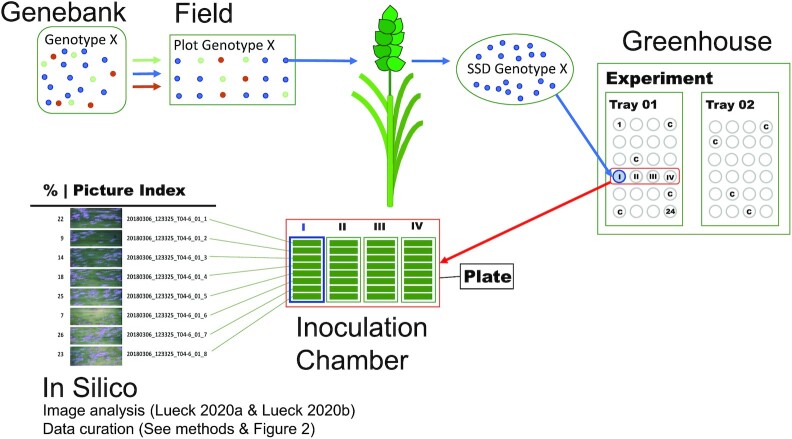
Schematic representation of the experimental design and the workflow of the Macrobot high-throughput powdery mildew phenotyping.

The maximum capacity of the inoculation tower of 12 plates defines the size of an independent experiment. Since each tray corresponds to 6 plates, 2 trays formed an independent experiment (see Fig. 1 for a graphical illustration). The inoculated plates were incubated for 6 days in an incubation chamber under standardized conditions (20°C, 60% relative humidity (RH), 16-hour photoperiod, 15 μE m^−2^ s^−1^). After this incubation time, images (3,296 × 2,472 pixels) were acquired using an RGB camera and stored in 24-bit TIFF format. Details of the used hardware are described in [19].

Based on the image data, the percentage of infected leaf area was determined by developing an open-source algorithm implemented in Python [18].

The independent experiments were linked by the susceptible cultivar KANZLER, which was also used for quality control. KANZLER was tested 4 times in each 24-slot tray (i.e., 8 times per experiment). In addition, to increase the reliability of the generated phenotypic data obtained, each genotype was tested in 2 or more independent experiments.

### Data curation of phenotypic data

To improve the quality of the data presented here, we developed and implemented an automatic stepwise quality control in the R environment [20]. First, we double-checked that the data structure and data format present in the recorded measurements and metadata corresponded with the actual design of phenotyping experiments. At this step, we controlled if lanes had a minimum number of 3 leaves and plates contained an exact number of 4 lanes. We also checked for errors in the label or lane detection of the automatic picture analysis and manual errors in the metadata. Data points that met these criteria were tested afterward for the presence of outliers at 3 different levels (steps).

In the first step, we tested the distribution of technical replicates of a measurement (up to 8 leaves per lane). We excluded outliers by using 1.5 times the interquartile distance as a threshold.

In the second step, we evaluated the data quality at the experiment level. There, we excluded whole experiments based on the infection of the susceptible control genotype KANZLER. The rationale behind this was that if the infection level of KANZLER was low, the inoculation of the experiment failed. To detect outliers here, we defined a threshold for the mean and maximal values of the control of each experiment by using the 1.5 interquartile distance or the infected leaf area again.

The third and final quality control step was based on the variance between the biological replicates (so the same genotype was tested in 2 different experiments). To do so, we fitted the same model as for best linear unbiased estimation (BLUE) and variance component estimation (see equation (1)) and defined a significant outlier threshold (*P* < 0.01) for the residuals of fitted genotypic means based on [21].

All computational methods were performed within the R environment [20] version 4.0.2. using R-Studio version 1.3.1056.

### BLUE and variance components estimation

To estimate the effect of the design parameters and correct the phenotypic values for those, we estimated the variance components and the BLUEs of the genotypes using the phenotypic data. BLUEs of the genotypes and variance components were estimated based on the curated data. For the estimation of variance components of the percentage of infected leaf area, we used the following linear mixed model [14]:


(1) \begin{eqnarray*} y{\rm{\ }} = {\rm{\ \mu \ }} + {\rm{\ }}genotype{\rm{\ }} + {\rm{\ }}experiment{\rm{\ }} + {\rm{\ }}tray\left( {experiment} \right) + {\rm{\ }}error, \end{eqnarray*}

where the common mean (*µ*) was treated as a fixed factor, whereas genotype, experiment, the tray nested within an experiment, and error effect were assumed as random factors. BLUEs were computed using the same model but assuming the genotype factor as a fixed effect. All linear mixed models were solved using the ASReml-R package version 4 [22].

The heritability was estimated as in the following equation:


(2) \begin{eqnarray*} {h}^2 = \ \frac{{\sigma _G^2}}{{\sigma _G^2 + \ \frac{{\sigma _e^2}}{R}}}, \end{eqnarray*}

where *σ_G_*^2^ is the genotypic variance, *σ_e_*^2^ is the residual variance, and $R$ represents the average number of replications (independent experiments) per genotype. The standard deviation of the heritability was estimated using a bootstrapping approach by performing 500 heritability estimations using random samples that contained 80% of the total number of genotypes.

### Genomic–phenomic data interoperability

The 7,398 defined PGR genotypes as well as elite cultivars were recently characterized using genotyping by sequencing (GBS) [16, 17]. Therefore, we also assessed the genomic–phenomic data interoperability based on the genomic best linear unbiased prediction (GBLUP) for leaf infections as an additional indicator of data quality.

For this prediction, we used a GBLUP model implemented in the kin.blup()-function, a wrapper for the mixed.solve()-function in the rrBLUP R package [23]. The fitted mixed model can be described as follows:


(3) \begin{eqnarray*} Y\ = {1}_n\ \mu + {\boldsymbol{Z}}g + e, \end{eqnarray*}

where $Y$ stands for a vector of trait values for $n$ genotypes, ${1}_n$ is a unit vector, $\mu $ corresponds to the population mean, ${\boldsymbol{Z}}$ indicates a design matrix linking the elements of g to $Y$, $g$ ($g\sim N( {0,\sigma _g^2{{\bf G}}} ))$ is a vector of random genotypic values, and $e$ ($e\sim N( {0,\sigma _e^2{\boldsymbol{I}}} )$) accounts for the random residual term. ${{\bf G}}$ represents an additive genomic relationship matrix based on the GBS marker and is calculated according to the first method of VanRaden [24]. ${\boldsymbol{I}}$ stands for an identity matrix, while $\sigma _g^2$ and $\sigma _e^2$ are the genotypic and error variance components, respectively. The assessment of the genomic–phenomic data interoperability was performed using a 5-fold cross-validation approach. The “fold” means in how many subparts we split the dataset: in our case, the dataset was randomly split into 5 parts in each cross-validation run. In more detail, the genomic and phenotypic data of the first 4 parts were used as training set to predict the (fifth) remaining part (called the test set) based only on the genomic data. Predictions were then compared with the observed phenotypes of the test set through correlation. The assignment of 4 parts to the training set and the fifth part to the test set was permutated in such a way that each subdivision served as the test set only once and was 4 times part of the training set. The mean correlation between predicted and observed values from the 5 different permutations was saved for each run. We performed 500 runs of this procedure.

## Data Description

The raw data described here as well as BLUEs, the raw images from the detached leaf assay, and the R script to import and curate the raw phenotypic data are available in the e!DAL-PGP-Repository [25] and can be directly accessed here [26]. In more detail, the repository contains the raw images of the individual measured leaves, the raw values of the infected leaf area predicted by the open-source Python implementation [18], and the curated, ready-to-use data in the form of BLUEs. We also provide the images of the whole plates.

To comply with the Findability, Accessibility, Interoperability, and Reuse (FAIR) principles of digital assets, the data were described according to the ISA-Tab format [27].

This includes an investigation file (“i_investigation.txt”) with general information about the conditions under which the data were produced and a description of the protocols used to generate and curate the presented data. The experimental conditions and design effects of the high-throughput assay are described in the corresponding study file (“s_GB2.0_MACRO_PM.txt”). The corresponding genotype identifiers to the previously published genotypic data for the population [16, 17] are also provided here. The assay file (“a_GB2.0_MACRO_PM.txt”) contains the predicted infected leaf area and the corresponding image identifier for each leaf value. In addition to that, we added the minimal, mean, and maximal average daily temperatures during the greenhouse period of each tested genotype to the data.

Specifically, the study file includes the effects of the experimental design of the Macrobot assay—namely, the experiment ID, the tray ID, and the replication number. Besides these, we provide the sowing, inoculation, and measuring dates. The “Source Name” is the accession number from the IPK Genebank Documentation System (GBIS) combined with an internal project number reflecting the defined seed (SSD in the case of PGR). Detecting mislabeling and duplicates, as well as correcting passport data, is a well-known challenge for genebanks worldwide [16]. For example, changes in the origin information or genotype names happen regularly. GBIS is therefore a constantly curated system and works with unique digital object identifiers (DOIs) to exactly trace back requested plant material to the source accessions and their information. We include GBIS DOIs as part of the data and encourage readers and users to use them instead of genotype names to get further information and request PGR for further research and breeding activities. In addition, SAMEA (SAM, BioSample accession; E, EBI; A, Assay Sample) numbers that link phenotypes to raw sequence reads are included. Sequence data can be accessed through SAMEA numbers [16, 17]. The “Sample Name” is a unique identifier, connecting the genotype ID in the study file with the raw phenotypic values in the assay file. It is also the name of the corresponding raw image.

In addition, the virulence pattern of the *B. graminis*f. sp.*tritici* isolate FAL 92315 is included in a CSV file. We also provide the phenotypic data as “raw_phenotype.csv,” which is used as input by the provided R-Script. We also give access to the BLUEs for the percentage of infected leaf area based on the curated raw data. These estimates are ready to use for different purposes (e.g., resistance donor selection, mapping approaches, or genomic prediction).

### Image data

The images generated by the Macrobot facility are the starting point for the analyses conducted. They were acquired using a Thorlabs 8050M-GE-TE camera (Newton, NJ, USA) at a resolution of 3,296 × 2,472 pixels with 365-nm (UV), 470-nm (blue), 530-nm (green), and 625-nm (red) peak wavelengths and white light back illumination—for more details, see [19]. The raw pictures of the whole plates are saved in a 24-bit TIFF format and are provided in the same repository. We cut out individual leaf positions from full-plate images to allow a datapoint-wise connection of phenotypic (percentage of infected leaf area) and picture data. Those images are also provided here in PNG format. Both sets of images have an expected resolution of 25 pixels/mm. The infected leaf area was determined on those images using the image analysis pipeline described in [18].

### Phenotypic data

**Figure 2:**
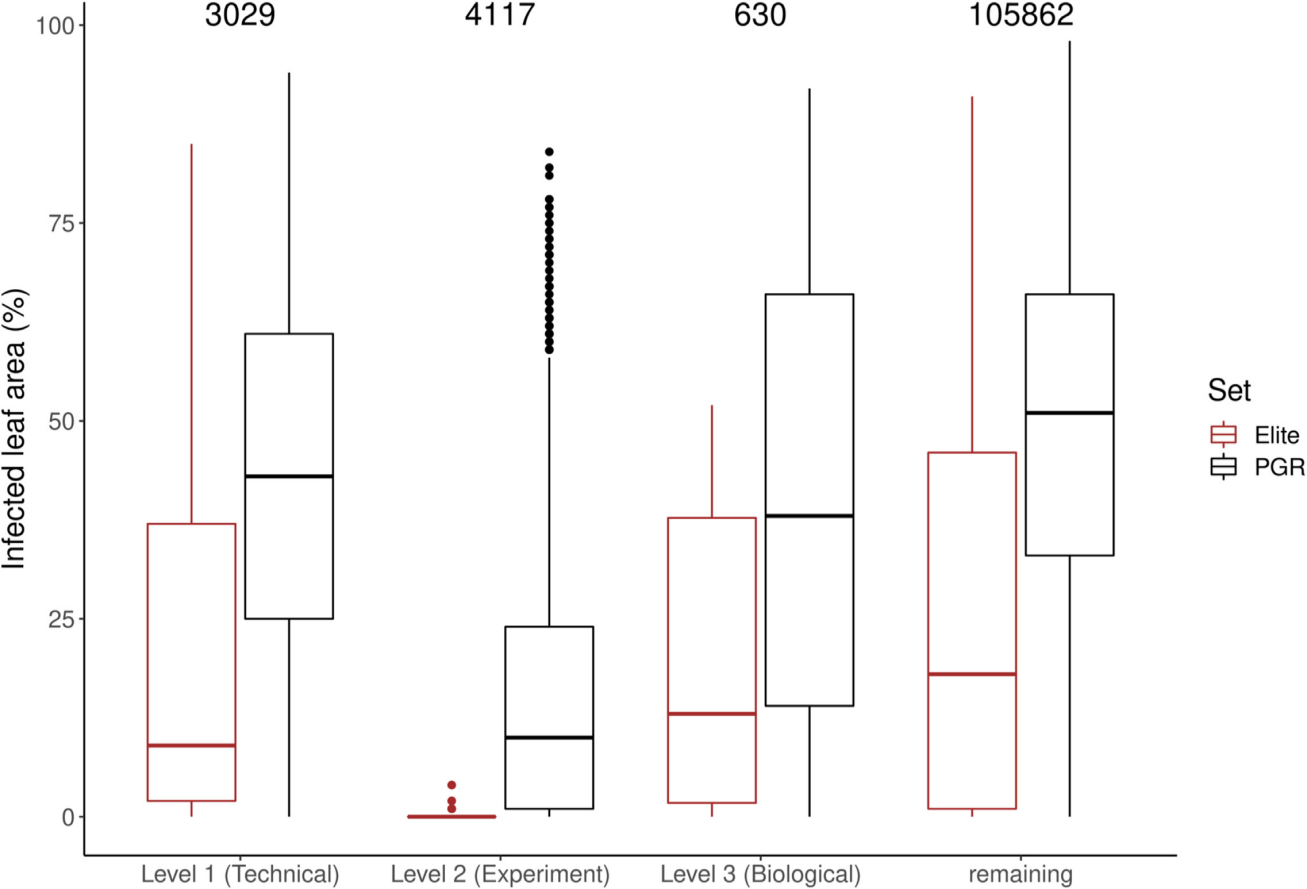
Distribution of the raw and curated (remaining) data that support the exclusion of extreme/unexpected datapoints at levels: (1) outlier(s) based on the technical replications of single genotypes; (2) outlier experiment(s) based on the infection level of the susceptible control genotype; and (3) outlier(s) based on the difference in infection levels of the biological replications of single genotypes. The numbers at the top of the graph indicate the number of datapoints in each category (for details, see section “Data curation of phenotypic data”).

The phenotypic data presented here concern the quantification of the infected leaf area. These data show the quantitative host–pathogen interaction in a controlled environment. Raw values range from 0% to 98% infected leaf area for the whole dataset (Fig. 2). PGR samples have a higher mean infection (47.25%) than the tested Elite Panel (24.14%), which presented in turn a slightly lower maximum infection value (91%). In total, we measured 113,638 leaves in 422 independent experiments (Table 1) connected through the control genotype KANZLER. On average, each genotype was tested in 1.96 experiments, with 7 genotypes tested up to 6 times and 290 tested only once. That a genotype, besides KANZLER, was unexpectedly tested in more than 2 independent experiments was due to few imparities during seed logistics. In the case of genotypes tested in no more than 1 experiment, this was mostly due to seed availability and/or germination issues. After outlier correction, 93.16% of the raw data were considered reliable and therefore used to compute BLUEs. We excluded 3,029 datapoints (measurements of leaves) (2.7%) due to outlier correction performed based on the technical replications. Due to failed experiments, we excluded 4,117 extra datapoints (i.e., 3.6% of the total data collected), while 630 datapoints (0.6% of the total data) were additionally excluded due to high differences between the biological replications.

**Table 1:** Variance components and heritability of the raw and curated phenotypic data. The factor “Experiment” refers to 422 independent experiments in which the data were generated. The factor “Tray” refers to the tray in which the plants were grown together.

	Raw data		Curated data	
Component	Estimation	SE	Estimation	SE
Experiment	199.85	14.63	158.89	12.14
Experiment: Tray	26.37	2.35	27.10	2.43
Genotype	138.46	3.43	151.78	3.65
Residual	137.11	1.87	129.89	1.83
Heritability	0.71		0.73	
SD	0.007		0.005	
Genotypes (PGR)	7,398		7,336	
Genotypes (Elite)	154		154	
Experiments	422		405	
Plates	4,887		4,694	
Lanes	14,830		14,177	
Leaves	113,638		105,862	

## Technical Validation

We used 2 criteria to evaluate the data quality presented here: first, heritability and, second, cross-validated genomic prediction.

**Figure 3:**
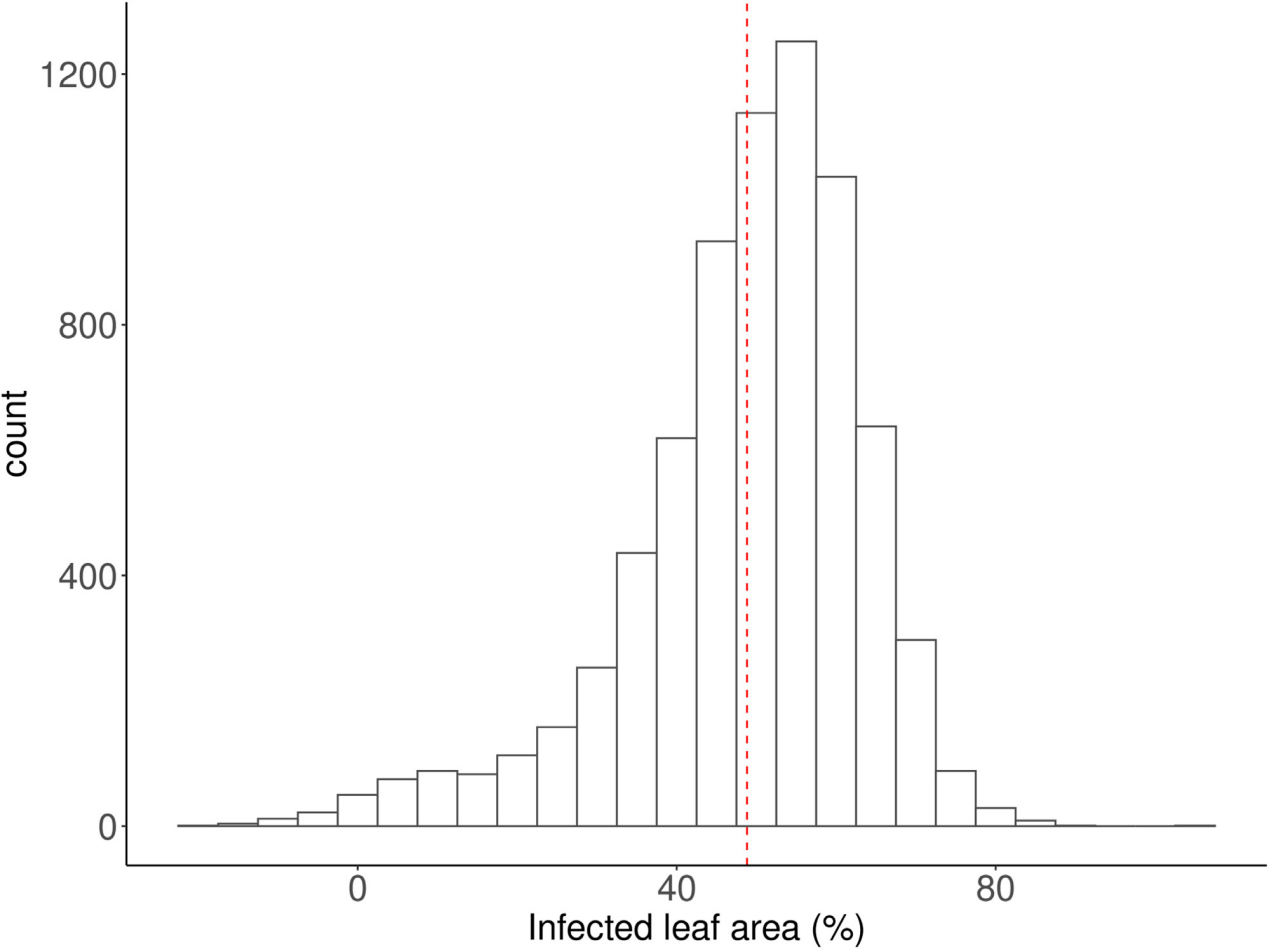
Histogram of the best linear unbiased estimations of the percentage of infected leaf area of 7,398 defined genotypes of plant genetic resources. The red dotted line represents the mean of the distribution.

The achieved heritability of the measured host–pathogen interaction was 0.73. Variance components analysis revealed a high effect of the experimental design on the raw phenotypes (Table 1). The performed data curation decreased the magnitude of the “Experiment” and residual effects while increasing, in turn, the variation proportion explained by the “Genotype” effect. This high heritability and the Gaussian-like distribution of the genotypic means or BLUEs (Fig. 3) support the quantitative nature of the resistance response against PM already at seedling stage.

To evaluate the genomic–phenomic data interoperability, we performed 500 runs of cross-validated genomic prediction. This analysis revealed a high mean prediction accuracy after data curation of 0.506 ± 0.004. In this regard, a 0.8% boost in accuracy could be attributed to the data curation steps.

## Summary and Outlook

We provide quantitative resistance phenotypes for 7,552 genotypes of winter wheat against *B. graminis*, causing PM infection at the seedling stage. Moreover, we showed that this quantification is possible and reliable using detached leaf assays—an approach traditionally used to characterize qualitative resistance. However, the method has also some of the limitations of detached leaf assays in seedlings in general:

It is mostly well suited for foliar diseases like leaf and stem rust, besides powdery mildew.

The weak to moderate correlation between our high-throughput data—obtained under artificially controlled conditions with a single isolate—and field data, fully relying on natural infections [14], indicates that our data should not be directly interpreted as field resistance. This is most likely because natural infections are the result of a diverse population of multiple pathotypes interacting with a changing environment and the crop. We therefore presume that testing different individual isolates, which are dominant in the current pathogen gene pool, could contribute to reduce this limitation.

The assessed quantitative resistance could provide crop plant protection effects by delaying the development of the pathogen population. All in all, the here presented dataset, in combination with already available genomic information and the possibility to connect the results from this assay with other studies using the PGR population of the IPK, will serve as a good base for an educated selection. Considering the diverse origins of the phenotyped plant genetic resources [16, 17], we expect to provide a valuable resource for breeders and scientists in different global regions.

## Supplementary Material

giad007_GIGA-D-22-00192_Original_Submission

giad007_GIGA-D-22-00192_Revision_1

giad007_GIGA-D-22-00192_Revision_2

giad007_GIGA-D-22-00192_Revision_3

giad007_Response_to_Reviewer_Comments_Original_Submission

giad007_Response_to_Reviewer_Comments_Revision_1

giad007_Response_to_Reviewer_Comments_Revision_2

giad007_Reviewer_1_Report_Original_Submission Antonin Dreiseitl -- 8/15/2022 Reviewed

giad007_Reviewer_2_Report_Original_Submission Feng Hui -- 8/16/2022 Reviewed

giad007_Reviewer_2_Report_Revision_1 Feng Hui -- 12/19/2022 Reviewed

giad007_Reviewer_3_Report_Original_Submission Rohit Mago, Ph.D. -- 8/18/2022 Reviewed

giad007_Reviewer_3_Report_Revision_1 Rohit Mago, Ph.D. -- 12/19/2022 Reviewed

giad007_Reviewer_4_Report_Original_Submission Chris Armit -- 8/30/2022 Reviewed

## Data Availability

The ready-to-use genotypic estimates (BLUEs) of infected leaf area, their supporting raw phenotypic data derived from detached leaf essay images in addition to their metadata (ISA-Tab format), and their corresponding raw (TIFF) and processed (PNG) image data sources were deposited at e!DAL-PGP under a CC0 license and can be accessed here [26]. In this repository, an R code to curate the raw phenotypic data and compute heritability and BLUEs is also available. For further details, please refer to the “Data Description” section.

## Abbreviations

BLUE: best linear unbiased estimation; DOI: digital object identifier; GBIS: Genebank Documentation System; GBLUP: genomic best linear unbiased prediction; GBS: genotyping by sequencing; IPK: Institute of Plant Genetics and Crop Plant Research; PGR: plant genetic resource; PM: powdery mildew; SSD: single-seed descent.
