# Supplementary material for: High-throughput imaging of powdery mildew resistance of the winter wheat collection hosted at the German Federal ex situ Genebank for Agricultural and Horticultural Crops
Source: Gigascience. 2023 Mar 3;12:giad007. doi: 10.1093/gigascience/giad007 (PMC9984986; doi:10.1093/gigascience/giad007)

GigaScience

High throughput imaging of powdery mildew resistance of the winter wheat collection hosted at the German Federal ex situ Genebank for Agricultural and Horticultural Crops

--Manuscript Draft--

**Manuscript Number:**	GIGA-D-22-00192
**Full Title:**	High throughput imaging of powdery mildew resistance of the winter wheat collection hosted at the German Federal ex situ Genebank for Agricultural and Horticultural Crops
**Article Type:**	Data Note
**Funding Information:**	BMBF (FKZ031B0184B) — Prof. Dr. Jochen C. Reif<br>BMBF (FKZ031B0184A) — Prof. Dr. Jochen C. Reif
**Abstract:**	Genebanks worldwide are transforming into bio-digital resource centres, providing not only access to the plant material itself but also to its phenotypic and genotypic information. Adding information for relevant traits will help boosting plant genetic resources' usage in breeding and research.<br>Resistance traits are vital for adapting our agricultural systems to future challenges. Here we provide phenotypic data for the resistance against Blumeria graminis, the causal agent of powdery mildew - a substantial risk to our agricultural production.<br>Using a modern high throughput phenotyping system, we infected and photographed a total of 113,638 wheat leaves of 7,398 winter wheat (Triticum aestivum L.) plant genetic resources of the German Federal Ex Situ Gene Bank for Agricultural and Horticultural Crops, Germany and 154 commercial genotypes. We quantified the resistance reaction captured by images and provide them here, along with the raw pictures.<br>This massive amount of phenotypic data combined with the already published genotypic data also provides a valuable and unique training dataset for the development of novel genotype-based predictions as well as mapping methods.
**Corresponding Author:**	Albert Wilhelm Schulthess Börgel<br>Leibniz-Institut für Pflanzengenetik und Kulturpflanzenforschung (IPK)<br>Seeland, Sachsen-Anhalt GERMANY
**Corresponding Author Secondary Information:**	
**Corresponding Author's Institution:**	Leibniz-Institut für Pflanzengenetik und Kulturpflanzenforschung (IPK)
**Corresponding Author's Secondary Institution:**	
**First Author:**	Valentin Hinterberger
**First Author Secondary Information:**	
**Order of Authors:**	Valentin Hinterberger<br>Dimitar Douchkov<br>Stefanie Lueck<br>Jochen C. Reif<br>Albert Wilhelm Schulthess Börgel
**Order of Authors Secondary Information:**	
**Additional Information:**	
**Question**	**Response**
Are you submitting this manuscript to a	No

Powered by Editorial Manager® and ProduXion Manager® from Aries Systems Corporation

special series or article collection?	
**Experimental design and statistics**<br><br>Full details of the experimental design and statistical methods used should be given in the Methods section, as detailed in our Minimum Standards Reporting Checklist. Information essential to interpreting the data presented should be made available in the figure legends.<br><br>Have you included all the information requested in your manuscript?	Yes
**Resources**<br><br>A description of all resources used, including antibodies, cell lines, animals and software tools, with enough information to allow them to be uniquely identified, should be included in the Methods section. Authors are strongly encouraged to cite Research Resource Identifiers (RRIDs) for antibodies, model organisms and tools, where possible.<br><br>Have you included the information requested as detailed in our Minimum Standards Reporting Checklist?	Yes
**Availability of data and materials**<br><br>All datasets and code on which the conclusions of the paper rely must be either included in your submission or deposited in publicly available repositories (where available and ethically appropriate), referencing such data using a unique identifier in the references and in the "Availability of Data and Materials" section of your manuscript.<br><br>Have you have met the above requirement as detailed in our Minimum Standards Reporting Checklist?	Yes

Powered by Editorial Manager® and ProduXion Manager® from Aries Systems Corporation

Manuscript

Click here to access/download;Manuscript;GB2.0_Data-Publ_Giga_20.07.2022.docx

Title

High throughput imaging of powdery mildew resistance of the winter wheat collection hosted at the *German Federal ex situ Genebank for Agricultural and Horticultural Crops*

Authors

Valentin Hinterberger[1](hinterberger@ipk-gatersleben.de),
Dimitar Douchkov[1] (douchkov@ipk-gatersleben.de),
Stefanie Lueck[1] (lueck@ipk-gatersleben.de),
Jochen C. Reif[1] (reif@ipk-gatersleben.de),
and Albert W. Schulthess[1,*] (schulthess@ipk-gatersleben.de)

Affiliations

[1] *Leibniz Institute of Plant Genetics and Crop Plant Research (IPK), D-06466, Seeland, Germany*

corresponding author: Albert W. Schulthess (schulthess@ipk-gatersleben.de)

Abstract

Genebanks worldwide are transforming into bio-digital resource centres, providing not only access to the plant material itself but also to its phenotypic and genotypic information. Adding information for relevant traits will help boosting plant genetic resources' usage in breeding and research.

Resistance traits are vital for adapting our agricultural systems to future challenges.

Here we provide phenotypic data for the resistance against *Blumeria graminis,* the causal agent of powdery mildew - a substantial risk to our agricultural production.

Using a modern high throughput phenotyping system, we infected and photographed a total of 113,638 wheat leaves of 7,398 winter wheat (*Triticum aestivum* L.) plant genetic resources of the *German Federal Ex Situ Gene Bank for Agricultural and Horticultural Crops, Germany* and 154 commercial genotypes. We quantified the resistance reaction captured by images and provide them here, along with the raw pictures.

This massive amount of phenotypic data combined with the already published genotypic data also provides a valuable and unique training dataset for the development of novel genotype-based predictions as well as mapping methods.

Background

Our agricultural system is facing one of the most significant upheavals in decades. In addition to uncertainties arising from ongoing climatic change and the ever-increasing demand for agricultural goods, the ecological impact of agricultural production is more than ever in the spotlight. The European "Farm to Fork Strategy" has set ambitious goals for a more sustainable agricultural production. One of these goals is to reduce pesticide use by 50% by 2030 (EU commission, 2020).

Fungicides form an important group of pesticides in cereal crops, which have been used regularly in intensive agriculture since the mid-1970s. The reasons why there is an urgent need to reduce the use of fungicides are manifold: harmful pesticide residues (Cabrera & Pastor, 2022), decreasing efficacy of active components due to pathogenic resistance (Lucas et al., 2015), and side effects on the environment and the crop (Calonne et al., 2011, Ullah et al., 2019) are just some of them.

There are many agronomical ways to reduce fungicide usage, e.g. precision farming (Zanin et al., 2022), improved crop rotation, changes in sowing date, and straw management. Growing resistant varieties is one of the easiest and most sustainable solutions for the farmer.

While easy to adopt for the farmer, breeding a stable resistant variety with excellent quality and high yield is a great challenge for breeders and phytopathologists.

Biotrophic pathogens like *Blumeria graminis,* the causal agent of powdery mildew (PM), show a rapid and strong response to deploying of new resistance mechanisms (Wolfe, 1984). The risk of pathogen populations adapting to resistance mechanisms correlates negatively with the diversity of the resistance mechanisms used (Lucas et al., 2015). Therefore, increasing resistance diversity is one way to stabilize yields without fungicides.

Providing donors for new, unused, or since a long-time abandoned resistance gene is one of the main purposes of genebanks like the *German Federal ex situ gene bank for Agricultural and Horticultural Crops, Germany*. However, finding useful plant genetic resources in thousands of accessions provided by genebanks is a challenge for breeders and scientists.

Therefore, we tested almost the entire IPK winter wheat collection for its resistance to PM. In this process, we infected and photographed a total of 113,638 wheat leaves of 7,398 accessions and 154 varieties used by farmers in Germany in the last decade. This data was obtained in a controlled environment and tested against the highly aggressive PM isolate FAL 92315.

The defined virulence spectrum of this isolate provides advantages over field data that usually rely on natural infections and much less controlled environmental conditions. Furthermore, it

is possible to reproduce this assay with additional lines in the future using the same environmental parameters and isolate. In combination with additional analysis using other isolates of *Blumeria graminis* it can be part of a genotype-by-genotype analysis elucidating host-pathogen interactions.

As a component of genome-wide mapping approaches, this data is a valuable source of information on donors for potentially novel resistance genes, as we recently have shown (Hinterberger et al., 2022). In addition, this dataset may help to develop or train new image analysis tools for images derived from detached leaf assays. Since the detached leaf assays are a standard method in phytopathology, cheap, easy, and repeatable quantification methods are highly desirable.

Methods

Plant material

The *Federal Ex Situ Genebank for Agricultural and Horticultural Crop Species*, Germany, hosted at the Leibniz Institute of Plant Genetics and Crop Plant Research (IPK) hosts more than 27,000 *Triticum aestivum* L. plant genetic resources (PGR) (Sharma et al., 2021). In this study we present phenotypic data for powdery mildew resistance of 7,398 PGR and 154 winter wheat varieties representing the cultivated varieties in Germany in the last decade (in the following denoted as the Elite Panel). In addition, a set of 929 additional genotypes (coded as Div_Set_1 - 929) were also tested in experiments but were not part of the study. Phenotypes of these additional genotypes were kept in the dataset to not disrupt the data structure and to allow proper correction for experimental design effects.

We used a "single seed descent" step to reduce genetic heterogeneity within accessions by sampling one genotype per homogenous accessions and two genotypes if accessions were heterogenous. In this step, we multiplied the seed samples in the field and selected one representative ear for further propagation (for details, see Schulthess et al., 2021). The genetically uniform PGR were then used for phenotyping and genotyping-by-sequencing (GBS). For the genotypes of the Elite Panel, seeds were obtained from local seed market providers therefore genetic homogeneity is assumed in this material.

High-throughput phenotyping of plant-pathogen interactions

The phenotypic data presented here was gathered using the Macrobot facility, a robotic platform performing high-throughput semi-automatic detached leaf assays (Lueck et al., 2020a,b). For the Macrobot assay, seedlings were grown in a tray with 6 × 4 slots in the greenhouse under standardized conditions. In each slot ten seedlings of the same genotype were grown. For the inoculation assay, leaves were cut from the second leaf of the 14-day-old seedlings. From those leaves two-cm-long segments were brought onto microtiter agar plates. Each plate consisted of four lanes, each with leaf segments from up to eight leaves per tested genotype These plates were then infected with highly virulent *Blumeria graminis f. sp. tritici* isolate FAL 92315 in a rotating platform by blowing spores from heavily infected leaves using a compressed air pistol.
The maximum capacity of the inoculation tower of twelve plates defines the size of an independent experiment. Since each tray corresponds to six plates, two trays formed an independent experiment (see figure 1 for a graphical illustration). The inoculated plates were incubated for six days in an incubation chamber under controlled conditions (20°C, 60% RH, 16 h photoperiod). After this incubation time, images (3296 × 2472 pixel) were acquired using

an RGB-Camera and stored in 16-bit TIFF format (details of the used hardware are described in Lueck et al., 2020b).
Based on the image data, the percentage of infected leaf area was determined by developing an open-source algorithm trained and implemented in Python (Lueck et al., 2020a).
The independent experiments were linked by the susceptible cultivar KANZLER, which was also used for quality control. KANZLER was tested four times in each 24-slot tray, i.e. eight times per experiment. In addition, to increase the reliability of the generated phenotypic data obtained, each genotype was tested in two or more independent experiments.

Data curation of phenotypic data

To improve the quality of the data presented here, we developed and implemented an automatic stepwise quality control in R environment (R Core Team, 2020). This script is also available within the data repository. First, we double-checked that the data structure and data format present in the recorded measurements and metadata correspond with the actual design of phenotyping experiments. At this step, we controlled if lanes had a minimum number of three leaves and plates contained an exact number of four lanes. We also checked for errors in the label or lane detection of the automatic picture analysis and manual errors in the metadata.
Data points that met these criteria were tested afterwards for their quality in three steps. In the first step, we tested the distribution of technical replicates of a measurement (up to eight leaves per lane). We excluded outliers by using 1.5 times the interquartile distance as a threshold.
In the second step, we evaluated the data quality at the experiment level. There, we excluded whole experiments based on the infection of the susceptible control genotype KANZLER. The rationale behind this was, that if the infection level of KANZLER is low, the inoculation of the experiment failed. To define outliers here, we defined a threshold for the mean and maximal values of the control of each experiment by using the 1.5 interquartile distance or the infected leaf area again.
The third and final quality control step was based on the variance between the biological replicates (so the same genotype was tested in two different experiments). To do so, we fitted the same model as for best linear unbiased estimation (BLUEs) and variance component estimation (see Equation 1) and defined a significant threshold (p-value < 0.01) for the residuals of fitted genotypic means based on Anscombe and Tukey (1963).
All computational methods were performed within R environment (R Core Team, 2020 version 4.0.2. using R-Studio version 1.3.1056).

Best linear unbiased estimation and variance components estimation

To estimate the effect of the design parameters and correct the phenotypic values for those, we estimated the variance components and the BLUEs of the genotypes using the phenotypic data. BLUEs of the genotypes and variance components were estimated based on the curated data. For the estimation of variance components of the percentage of infected leaf area, we used the following linear mixed model (Hinterberger et al. 2022):

$$y = \mu + genotype + experiment + tray(experiment) + error \tag{1}$$

where the common mean (μ) was treated as a fixed factor, whereas genotype, experiment, the tray nested within an experiment, and error effect were assumed as random factors. BLUEs were computed using the same model but assuming the genotype factor as a fixed effect. All linear mixed models were solved using the ASReml-R package Version 4 (Butler et al., 2017).
The heritability was estimated as in the following equation:

$$h^2 = \frac{\sigma_G^2}{\sigma_G^2 + \frac{\sigma_e^2}{R}} \quad (2)$$

where $\sigma_G{}^2$ is the genotypic variance, $\sigma_e{}^2$ is the residual variance while R represents the average number of replications per genotype. The standard deviation of the heritability was estimated by performing 500 heritability estimations using random samples that contained 80% of the total number of genotypes.

Genomic-phenomic data interoperability

In addition to the heritability as an indicator of data quality, we also assessed the genomic-phenomic data interoperability based on the genomic best linear unbiased prediction (GBLUP). We used the publically available (Schulthess et al., 2021) single nucleotide polymorphisms (SNPs) data generated in a genotyping-by-sequencing approach.
For this prediction, we used a GBLUP model implemented in the kin.blup()-function, a wrapper for the mixed.solve()-function in the rrBLUP-Package (Endelman, 2011). The fitted mixed model can be described as follows:

$$Y = \mathbf{1}_n\mu + \boldsymbol{Z}g + e \quad (3)$$

Where Y stands for a vector of trait values for n genotypes, $\mathbf{1}_n$ is a unit vector, μ stands for the population mean, $\boldsymbol{Z}$ stands for a design matrix linking the elements of g to Y, g ($g \sim N(0, \sigma_g^2\mathbf{G})$) stands for a vector of random genotypic values and e ($e \sim N(0, \sigma_e^2\boldsymbol{I})$) is the random residual term. $\mathbf{G}$ represents an additive genomic relationship matrix, based on GBS marker (coded as -1,0,1), here the first method of VanRaden (VanRaden, 2008) was used. $\boldsymbol{I}$ stands for an identity matrix while σ_g^2 and σ_e^2 are the genotypic and error variance components, respectively.
The prediction was performed by randomly splitting the dataset into 5 parts of similar size. Each of those parts were than predicted using the other 4 parts of the dataset to train the model. As accuracy measurement we used the correlation of testset prediction and the measured values. Out of those 5 predictions the mean value was saved. We performed 500 runs of this procedure.

Data description

The here described data, the raw pictures from the detached leaf assay, and the R-Script to import and curate the raw phenotypic data is available in the e!DAL-PGP-Repository (Arend et

al., 2014 and can be directly accessed here (https://doi.ipk-gatersleben.de/DOI/dc5316a5-aad7-423b-9ce7-2d972acc0ac8/182f2ae0-6879-4f9a-980a-2c217c8e8c6b/2/1847940088) *(Note: This is not the final DOI, it will be generated as soon as the review process is completed)*

The repository contains the raw images of the individual measured leaves, the raw values of the predicted infected leaf area, and the curated, ready-to-use data in the form of BLUEs. To comply with the FAIR principles, the data were described according to the ISA-Tab format (Sansone et al., 2012).

This includes an investigation file ("i_investigation.txt") with general information about the conditions under which the data were created and a description of the protocols used to generate and curate the presented data. The experimental conditions and design effects of the high throughput assay are described in the corresponding study file ("s_GB2.0_MACRO_PM.txt"). The corresponding genotype identifiers to the previously published genotypic data for the population (Schulthess et al., 2021) are also provided here. The assay file ("a_GB2.0_MACRO_PM.txt") contains the predicted infected leaf area and the corresponding image identifier for each leaf value.

Specifically, the study file includes the effects of the experimental design of the Macrobot assay, namely the Experiments ID, the Tray ID, and the Replication Nr. Besides these, we provide the sowing, inoculation and measuring dates. The "Source Name" is the Accession number from the IPK Genebank Documentation System (GBIS) combined with an internal project number reflecting the derived SSD line from the accession. We also added the GBIS DOI, a direct link to more information about the accession from the Genebank Information System at the IPK. This link also allows direct ordering of accessions of interest for scientific and breeding purposes. In addition, SAMEA (SAM, BioSample accession; E, EBI; A, Assay Sample) numbers that link phenotypes to raw sequence reads are included. Sequence data can be accessed through SAMEA numbers at https://www.ebi.ac.uk/biosamples/.

The "Sample Name" is a unique identifier, connecting the genotype ID in the study-file with the raw phenotypic values in the assay file. It is also the name of the corresponding raw image. In addition, we also provide the phenotypic data in a .csv file "raw_phenotype.csv", which is used by the provided R-Script.

We also give access to the BLUEs for the percentage of infected leaf area based on the curated raw data. These estimates are ready-to-use for different purposes (e.g., mapping approaches of resistance donor selection).

Image data

The images generated by the Macrobot facility are the starting point for the analyses conducted. They were acquired using a Thorlabs 8050M-GE-TE camera at a resolution of 3,296 × 2,472px with 365 nm (UV), 470 nm (blue), 530 nm (green), and 625 nm (red) peak wavelengths, and white light back illumination (for more details, see Lueck et al., 2020b).

The raw pictures of the whole plates are saved in 16-bit TIFF format. We separated the images of individual leaves to allow a datapoint-wise connection of phenotypic data and picture data. Those images are provided here in PNG-format.

The infected leaf area was predicted on those images using the image analysis pipeline described in Lueck et al. 2020a.

Phenotypic data

The phenotypic data presented here concern the quantification of the infected leaf area. These data show the quantitative host-pathogen interaction in a controlled environment. Raw values range from 0 to 98 % infected leaf area with a mean for the whole dataset of 48.16 % (Figure 2). We observed a lower mean for the tested Elite Panel (31.87 %) and a slightly lower maximum value (94 %). In total, we measured 113,638 leaves in 422 independent experiments

(Table 1) connected through the control genotype KANZLER. On average, each genotype was tested in 1.95 experiments, with some tested up to 6 times and others tested only once. After outlier correction, 93.4% of the raw data were considered reliable and therefore used to compute BLUEs.

Technical validation

We used two criteria to evaluate the data quality presented here: first, heritability, and second, cross-validated genomic prediction. The achieved heritability of the measured host-pathogen interaction was 0.75. Variance components analysis revealed a high effect of the experimental design on the phenotype. (Table 1). The performed data curation decreased the magnitude of the "Experiment" and residual effects increased in turn the variation proportion explained by the "Genotype" effect.

To evaluate the genomic-phenomic- data-interoperability, we performed 500 runs of cross-validated genomic prediction. This analysis revealed a high prediction accuracy after data curation 0.507±0.004. In this regard, a 0.4% boost in accuracy could be attributed to the data curation steps.

Code availability

All computational methods were performed within R environment (R Core Team 2020 version 4.0.2. using R-Studio version 1.3.1056). The Code to import and curate the data ("GB2.0_Macro_PM_15.06.2022.R") is available in the same e!DAL repository (https://doi.ipk-gatersleben.de/DOI/dc5316a5-aad7-423b-9ce7-2d972acc0ac8/182f2ae0-6879-4f9a-980a-2c217c8e8c6b/2/1847940088)

(Note: This is not the final DOI, it will be generated as soon as the review process is completed)

Acknowledgements

The experimental work was supported by the German Federal Ministry of Education and Research within the GeneBank2.0 Project (Grant Nos. FKZ031B0184B and FKZ031B0184A) and supported by the German Plant Phenotyping Network (DPPN) (FKZ 031A053).

We would like to acknowledge the following colleagues for the valuable technical help of in performing the experiments (in alphabetical order): Md. Al Mamum, Sonja Alner, Evangeline G. Avogadro, Federico Barbier, Ruben Betz, Gabriele Brantin, Bettina Brückner, Alessia De Matteis, Deniz Demirhan, Birgit Dubsky, André Fessel, Lena Gaczensky, Christin-Sophie Gäde, Armand Garcia, Sonja Gentz, Kathrin Gramel-Koch, Bettina Kersten, Andrea Kunze , Martina Kühne, Gabriele Lange, Ingrid Marscheider, Liana Münchhoff, Jelena Perovic, Linda Ries, Gabriele Stentzel, Julia Sturz, Jacqueline Templer, Claudia Voigt and Ellen Weiss

We thank Moritz Lell for his bioinformatic support and the many fruitful discussions.

Author contributions

AWS and JCR designed the study; DD generated phenotypic data; SL performed the image analysis, VH curated the data, performed the analyses, and wrote the manuscript with the input of all other authors.

Competing interests

The authors declare no conflict of interest.

Figures

Figure 1: Schematic representation of the experimental design and the workflow of the Macrobot high throughput powdery mildew phenotyping (modified from Hinterberger et al., 2022)

Figure 2: Distribution of the raw and curated data that supports the exclusion of extreme/unexpected datapoints at levels: (1): Outlier(s) based on the technical replications of single genotypes; (2) Outlier experiment(s) based on the infection level of the susceptible control genotype; (3) Outlier(s) based on the difference in infection levels of the biological replications of single genotypes. The numbers at the top of the graph indicate the number of datapoints in each category (For details, see chapter: Data Curation of phenotypic data)

Tables

Table 1: Variance components and Heritability of the raw and curated phenotypic data. The factor "Experiment" refers to 446 independent experiments in which the data was generated. The Factor "Tray" refers to the tray in which the plants were grown together

	Raw Data		Curated Data	
**Component**	**Estimation**	**SE**	**Estimation**	**SE**
Experiment	198.89	14.52	157.58	11.99
Experiment:Tray	25.46	2.29	26.03	2.35
Genotype	159.77	3.73	172.69	3.92
Residual	140.16	1.89	131.26	1.83
Heritability	0.73		0.75	
SD	0.005		0.005	
Genotypes(PGR)	7,398		7,336	
Genotypes(Elite)	154		154	
Experiments	422		405	
Plates	4,887		4,694	
Lanes	14,830		14,177	
Leaves	113,638		105,862	

References:

[1] European commission. A Farm to Fork Strategy for a fair healthy and environmentally-friendly food system; 2020; CELEX:52020DC0381.

[2] Cabrera LC, Pastor PM. The 2020 European Union report on pesticide residues in food. EFSA Journal. 2022; doi: 10.2903/j.efsa.2022.7215

[3] Lucas JA, Hawkins JN, Fraaije BA. The Evolution of Fungicide Resistance. In: Sariaslani S, Gadd GM, editors. Advances in Applied Microbiology. Academic Press; 2015. P 29-92; doi: 10.1016/bs.aambs.2014.09.001

[4] Calonne M, Fontaine J, Debiane D, et al. Side effects of the sterol biosynthesis inhibitor fungicide, propiconazole, on a beneficial arbuscular mycorrhizal fungus. Communications in Agricultural and Applied Biological Sciences. 2011

[5] Ullah MR, Dijkstra FA. Fungicide and Bactericide Effects on Carbon and Nitrogen Cycling in Soils: A Meta-Analysis. Soil Systems. 2019; doi:10.3390/soilsystems3020023

[6] Zanin ARA, Neves DC, Teodoro LPR et al. Reduction of pesticide application via real-time precision spraying. Sci Rep. 2022; doi:10.1038/s41598-022-09607-w

[7] Wolfe MS, Trying to understand and control powdery mildew. Plant pathology. 1984

[8] Sharma S, Schulthess AW, Bassi FM, et al. Introducing Beneficial Alleles from Plant Genetic Resources into the Wheat Germplasm. Biology. 2021; doi:10.3390/biology10100982

[9] Schulthess AW, Kale SM, Liu F, et al. GiPS: Genomics-informed parent selection uncovers the breeding value of wheat genetic resources. bioRxiv. 2021; doi: 10.1101/2021.12.15.472759.

[10] Lueck S, Beukert U, Douchkov D. BluVision Macro - a software for automated powdery mildew and rust disease quantification on detached leaves. Journal of Open Source Software. 2020a; doi:10.21105/joss.02259

[11] Lueck S, Strickert M, Lorbeer M, et al. "Macrobot": An Automated Segmentation-Based System for Powdery Mildew Disease Quantification. Plant Phenomics. 2020b; doi:10.34133/2020/5839856

[12] R Core Team. R: A language and environment for statistical computing. R Foundation for Statistical Computing, Vienna, Austria. 2020; URL: https://www.R-project.org/.

[13] Anscombe FJ, Tukey JW. The examination and analysis of residuals. Technometrics. 1963

[14] Hinterberger V, Douchkov D, Lueck S, et al. Mining for New Sources of Resistance to Powdery Mildew in Genetic Resources of Winter Wheat. Front Plant Sci. 2022; doi:10.3389/fpls.2022.836723

[15] Butler DG, et al. ASReml-R Reference Manual Version 4. VSN International Ltd, Hemel Hempstead, HP1 1ES, UK; 2017

[16] Endelman JB. Ridge regression and other kernels for genomic selection with r package rrblup. The Plant Genome Journal. 2011; doi:10.3835/plantgenome2011.08.0024

[17] VanRaden PM. Efficient Methods to Compute Genomic Predictions. Journal of Dairy Science. 2008; doi:10.3168/jds.2007-0980.

[18] Arend D, Lange M, Chen J, et al. e!DAL - a framework to store, share and publish research data. BMC Bioinformatics. 2014; doi:10.1186/1471-2105-15-214

[19] Sansone SA, Rocca-Serra P, Field D, et al. Toward interoperable bioscience data. Nature Genetics. 2012; doi:10.1038/ng.1054

Figure 2

Click here to access/download;Figure;Figure 2.jpg

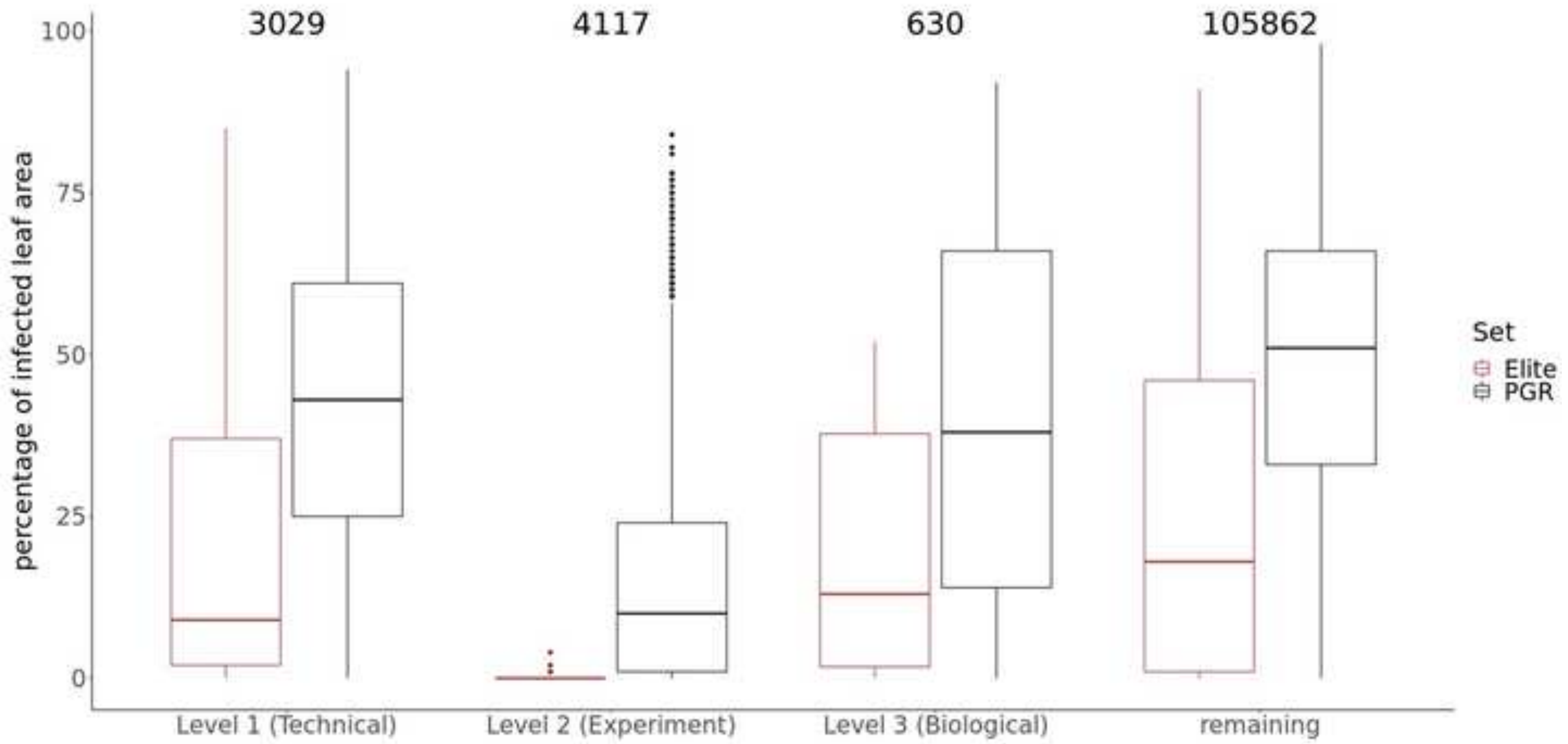

Figure 1

Click here to access/download;Figure;Figure 1.jpg

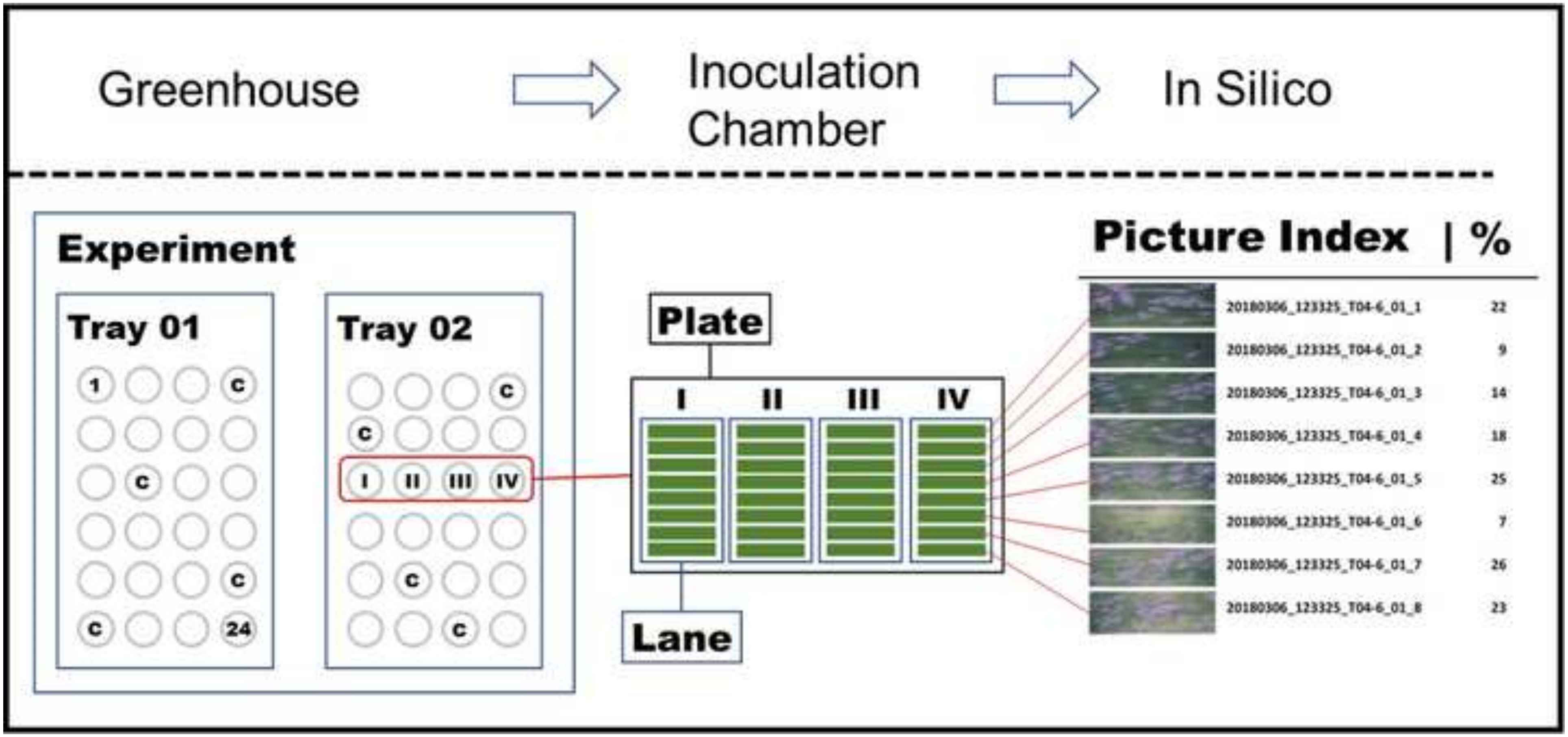

Supplement: giad007_GIGA-D-22-00192_Original_Submission [file giad007_giga-d-22-00192_original_submission.pdf]
