# Supplementary material for: High-throughput imaging of powdery mildew resistance of the winter wheat collection hosted at the German Federal ex situ Genebank for Agricultural and Horticultural Crops
Source: Gigascience. 2023 Mar 3;12:giad007. doi: 10.1093/gigascience/giad007 (PMC9984986; doi:10.1093/gigascience/giad007)
Supplement: giad007_GIGA-D-22-00192_Revision_1 [file giad007_giga-d-22-00192_revision_1.pdf]

GigaScience

High throughput imaging of powdery mildew resistance of the winter wheat collection hosted at the German Federal ex situ Genebank for Agricultural and Horticultural Crops

--Manuscript Draft--

**Manuscript Number:**	GIGA-D-22-00192R1
**Full Title:**	High throughput imaging of powdery mildew resistance of the winter wheat collection hosted at the German Federal ex situ Genebank for Agricultural and Horticultural Crops
**Article Type:**	Data Note
**Funding Information:**	BMBF (FKZ031B0184B) — Prof. Dr. Jochen C. Reif<br>BMBF (FKZ031B0184A) — Prof. Dr. Jochen C. Reif
**Abstract:**	Genebanks worldwide are transforming into bio-digital resource centres, providing not only access to the plant material itself but also to its phenotypic and genotypic information. Adding information for relevant traits will help boosting plant genetic resources' usage in breeding and research. Resistance traits are vital for adapting our agricultural systems to future challenges. Here we provide phenotypic data for the resistance against Blumeria graminis, the causal agent of powdery mildew - a substantial risk to our agricultural production. Using a modern high-throughput phenotyping system, we infected and photographed a total of 113,638 wheat leaves of 7,505 winter wheat (Triticum aestivum L.) plant genetic resources of the German Federal Ex Situ Genebank for Agricultural and Horticultural Crops and 154 commercial genotypes. We quantified the resistance reaction captured by images and provide them here, along with the raw images. This massive amount of phenotypic data combined with already published genotypic data also provides a valuable and unique training dataset for the development of novel genotype-based predictions as well as mapping methods.
**Corresponding Author:**	Albert Wilhelm Schulthess Börgel<br>Leibniz-Institut für Pflanzengenetik und Kulturpflanzenforschung (IPK)<br>Seeland, Sachsen-Anhalt GERMANY
**Corresponding Author Secondary Information:**	
**Corresponding Author's Institution:**	Leibniz-Institut für Pflanzengenetik und Kulturpflanzenforschung (IPK)
**Corresponding Author's Secondary Institution:**	
**First Author:**	Valentin Hinterberger
**First Author Secondary Information:**	
**Order of Authors:**	Valentin Hinterberger<br>Dimitar Douchkov<br>Stefanie Lueck<br>Jochen C. Reif<br>Albert Wilhelm Schulthess Börgel
**Order of Authors Secondary Information:**	
**Response to Reviewers:**	Dear Hans,<br><br>You will find our Cover Letter and detailed answers to the comments of reviewers in the attached document file "Cover_letter.docx" labelled as "Cover letter and response to reviewers". We do not know if all reviewers have access to the original files or only to the PDF which is automatically generated by the editorial manager system.

Powered by Editorial Manager® and ProduXion Manager® from Aries Systems Corporation

	Therefore, we included two versions of our manuscript: in the main (first) file all changes performed during revision are "accepted", hence not be seen, but readability is improved. In the second file (included at the end of the PDF, after our answers to the reviewers), all changes can be seen for transparency. We hope that this can make the review process more efficient.<br><br>Best regards,<br>Albert W. Schulthess
**Additional Information:**	
**Question**	**Response**
Are you submitting this manuscript to a special series or article collection?	No
**Experimental design and statistics**<br><br>Full details of the experimental design and statistical methods used should be given in the Methods section, as detailed in our Minimum Standards Reporting Checklist. Information essential to interpreting the data presented should be made available in the figure legends.<br><br>Have you included all the information requested in your manuscript?	Yes
**Resources**<br><br>A description of all resources used, including antibodies, cell lines, animals and software tools, with enough information to allow them to be uniquely identified, should be included in the Methods section. Authors are strongly encouraged to cite Research Resource Identifiers (RRIDs) for antibodies, model organisms and tools, where possible.<br><br>Have you included the information requested as detailed in our Minimum Standards Reporting Checklist?	Yes
**Availability of data and materials**<br><br>All datasets and code on which the conclusions of the paper rely must be either included in your submission or	Yes

Powered by Editorial Manager® and ProduXion Manager® from Aries Systems Corporation

deposited in publicly available repositories (where available and ethically appropriate), referencing such data using a unique identifier in the references and in the "Availability of Data and Materials" section of your manuscript.<br><br>Have you have met the above requirement as detailed in our Minimum Standards Reporting Checklist?	

Revised Manuscript (Plain text)

Click here to access/download;Manuscript;GB2.0_Data-Publ_Giga_revised.docx

Title

High throughput imaging of powdery mildew resistance of the winter wheat collection hosted at the *German Federal ex situ Genebank for Agricultural and Horticultural Crops*

Authors

Valentin Hinterberger[1](hinterberger@ipk-gatersleben.de),
Dimitar Douchkov[1] (douchkov@ipk-gatersleben.de),
Stefanie Lueck[1] (lueck@ipk-gatersleben.de),
Jochen C. Reif[1] (reif@ipk-gatersleben.de),
and Albert W. Schulthess[1,*] (schulthess@ipk-gatersleben.de)

Affiliations

[1] *Leibniz Institute of Plant Genetics and Crop Plant Research (IPK), D-06466, Seeland, Germany*

corresponding author: Albert W. Schulthess (schulthess@ipk-gatersleben.de)

Abstract

Genebanks worldwide are transforming into bio-digital resource centres, providing not only access to the plant material itself but also to its phenotypic and genotypic information. Adding information for relevant traits will help boosting plant genetic resources' usage in breeding and research. Resistance traits are vital for adapting our agricultural systems to future challenges. Here we provide phenotypic data for the resistance against *Blumeria graminis,* the causal agent of powdery mildew - a substantial risk to our agricultural production. Using a modern high-throughput phenotyping system, we infected and photographed a total of 113,638 wheat leaves of 7,505 winter wheat (*Triticum aestivum* L.) plant genetic resources of the *German Federal Ex Situ Genebank for Agricultural and Horticultural Crops* and 154 commercial genotypes. We quantified the resistance reaction captured by images and provide them here, along with the raw images. This massive amount of phenotypic data combined with already published genotypic data also provides a valuable and unique training dataset for the development of novel genotype-based predictions as well as mapping methods.

Background

Our agricultural system is facing one of the most significant upheavals in decades. In addition to uncertainties arising from ongoing climatic change and the ever-increasing demand for agricultural goods, the ecological impact of agricultural production is more than ever in the spotlight. In this context, the European "Farm to Fork Strategy" has set ambitious goals for a more sustainable agricultural production. One of these goals is to reduce pesticide use by 50% by 2030 (EU commission, 2020). Fungicides form an important group of pesticides in cereal crops, which have been used regularly in intensive agriculture since the mid-1970s. The reasons why there is an urgent need to reduce the use of fungicides are manifold: harmful pesticide residues (Cabrera and Pastor, 2022), decreasing efficacy of active components due to pathogenic resistance (Lucas et al., 2015), and side effects on the environment and the crop (Calonne et al., 2011, Ullah et al., 2019) are just some of them.

There are many agronomical ways to reduce fungicide usage, e.g. precision farming (Zanin et al., 2022), improved crop rotation, changes in sowing date, and straw management. Growing resistant varieties is one of the easiest and most sustainable solutions for the farmer. While easy to adopt for the farmer, breeding a stable resistant variety with excellent quality and high yield is a great challenge for breeders and phytopathologists. The past decades have shown continuous cycles of a “Boom and Bust” pattern in resistance development - new major qualitative resistance mechanisms are identified and heavily used in agriculture. This has led to a strong selection pressure on the pathogen population and an inevitable break down of the resistance by population shift and mutations (McDonald and Linde, 2002, Wolfe, 1984). Especially biotrophic pathogens like *Blumeria graminis,* the causal agent of powdery mildew (PM), show a rapid and strong response to deploying of new resistance mechanisms (Wolfe, 1984). In this context, the risk of pathogen populations adapting to resistance mechanisms can be delayed by increasing diversity of the resistance mechanisms in cultivars and relying on quantitative resistance provided by the additive effect of several minor resistance genes (Lucas et al., 2015, McDonald and Linde 2002).

Providing donors for new, unused, or since a long-time abandoned resistance genes is one of the main purposes of genebanks like the *German Federal Ex situ Genebank for Agricultural and Horticultural Crops*. The great challenge for breeders and scientists lies here on finding useful plant genetic resources (PGR) among thousands of genebank accessions. In order to make these informed prebreeding decisions possible, we have tested almost all of IPK's winter wheat (*Triticum aestivum* L.) collection for its quantitative resistance to PM by combining high-throughput imaging of detached leaf assays and a machine-based quantification of the percentage of infected leaf area. In this process, we infected and photographed a total of 113,638 wheat leaves of 7,505 accessions and 154 varieties used by farmers in Germany during the last decades. This data was obtained in a controlled environment at the seedling stage and using the highly virulent PM isolate FAL 92315. Under this highly controlled setup and provided a

strong genotypic effect of host plants, fungal growth could be attributed to a quantitative resistance response of genotypes. Such a reliable association would most likely not be possible based on field data that rely on natural infections and much less controlled environmental conditions.

Detached leaf assays are a standard method in phytopathology to assess plant resistance in a cheap, fast, easy, and repeatable manner (Torp et al., 1978). They are traditionally performed to measure the qualitative resistance response at the seedling stage of plants. However, there is evidence for quantitative resistance mechanisms in seedlings. For example, *Lr34* confers partial resistance already at the seedling stage (Rubiales and Niks, 1995) while some *SWEET* genes have been associated to quantitative susceptibility in seedlings (Chen et al., 2014, Gupta, 2020). Some of those quantitative or partial resistance mechanisms have a delaying (latency) effect on the development of the pathogen, resulting in longer reproduction cycles and a reduced spore production by the pathogen (Niks et al., 2015). We therefore investigated the plausibility of capturing latency mechanisms of quantitative resistance against PM at the seedling stage in a detached leaf assay setup applied at a large-scale to genebank material.

The here presented data can be further extended with additional untested plant material by using the same environmental parameters and isolate. In addition, this dataset may help to develop or train new image analysis tools for images derived from detached leaf assays. In combination with additional analysis using other isolates of *Blumeria graminis*, it can be part of a genotype-by-genotype analysis elucidating host-pathogen interactions. As a component of genome-wide mapping approaches, this data is a valuable source of information on donors for potentially novel resistance genes, as we recently have shown (Hinterberger et al., 2022). We expect that our quantitative resistance data contribute to the discovery of basal resistance mechanisms that provide a more durable crop protection in the future.

Methods

Plant material

The *German Federal Ex Situ Genebank for Agricultural and Horticultural Crop Species* located at the Leibniz Institute of Plant Genetics and Crop Plant Research (IPK) hosts more than 27,000 wheat PGR of the *Triticum sp.* genus (Sharma et al., 2021). In this study we present phenotypic data for powdery mildew resistance of 7,505 wheat PGR and 154 winter wheat varieties representing the cultivated varieties in Germany in the last decade (in the following denoted as the Elite Panel). In addition, a set of 929 additional genotypes (coded as Div_Set_1 – 929) were also tested in experiments but were not part of the study. Phenotypes of these additional genotypes were kept in the dataset to not disrupt the data structure and to allow proper correction for experimental design effects.

During field multiplication of genebank material, we used a "single seed descent" (SSD) step to obtain defined seeds (for details, see Schulthess et al., 2022). This was achieved by bagging one representative ear for each of 7,502 homogenous accessions and two ears in case of only three accessions, which we identified as clearly heterogenous based on the morphological appearance of plants within each accession. These defined seeds were also used for genotyping-by-sequencing (GBS) in a companion study (Schulthess et al., 2022). For the genotypes of the Elite Panel, defined seeds were obtained from local seed market providers.

High-throughput phenotyping of plant-pathogen interactions

The phenotypic data presented here was gathered using the Macrobot facility, a robotic platform performing high-throughput semi-automatic detached leaf assays (Lueck et al., 2020a,b). For the Macrobot assay, seedlings from defined seeds were grown in trays with 6 × 4 slots in the greenhouse under standardized conditions. In each slot ten seedlings of the same genotype were grown. For the inoculation assay, a leaf segment was cut from the second leaf of the 14-day-old seedlings. We cut the middle part of the leaf because early trials evidenced that the base of the leaf is more susceptible to

powdery mildew, while the tip is more resistant (data not shown). The two-cm-long leaf segments were brought onto microtiter agar plates. Each plate consisted of four lanes, each with leaf segments from up to eight leaves per tested genotype. These plates were then infected with highly virulent *Blumeria graminis f. sp. tritici* isolate FAL 92315 (please see Supplementary Table 1 for the respective virulence/avirulence spectrum) in a rotating platform by blowing spores from heavily infected leaves using a compressed air pistol.

The maximum capacity of the inoculation tower of twelve plates defines the size of an independent experiment. Since each tray corresponds to six plates, two trays formed an independent experiment (see Figure 1 for a graphical illustration). The inoculated plates were incubated for six days in an incubation chamber under standardized conditions (20°C, 60% RH, 16 h photoperiod, $15\ \mu E\ m^{-2}\ s^{-1}$). After this incubation time, images (3296 × 2472 pixel) were acquired using an RGB-Camera and stored in 16-bit TIFF format (details of the used hardware are described in Lueck et al., 2020b).

Based on the image data, the percentage of infected leaf area was determined by developing an open-source algorithm implemented in Python (Lueck et al., 2020a).

The independent experiments were linked by the susceptible cultivar KANZLER, which was also used for quality control. KANZLER was tested four times in each 24-slot tray, i.e. eight times per experiment. In addition, to increase the reliability of the generated phenotypic data obtained, each genotype was tested in two or more independent experiments.

Data curation of phenotypic data

To improve the quality of the data presented here, we developed and implemented an automatic stepwise quality control in the R environment (R Core Team, 2020). First, we double-checked that the data structure and data format present in the recorded measurements and metadata correspond with the actual design of phenotyping experiments. At this step, we controlled if lanes had a minimum number of three leaves and plates contained an exact number of four lanes. We also checked for errors in the label or lane detection of the automatic picture analysis and manual errors in the metadata. Data points that met these criteria were tested afterwards for the presence of outliers at three different levels (steps):

In the first step, we tested the distribution of technical replicates of a measurement (up to eight leaves per lane). We excluded outliers by using 1.5 times the interquartile distance as a threshold.

In the second step, we evaluated the data quality at the experiment level. There, we excluded whole experiments based on the infection of the susceptible control genotype KANZLER. The rationale behind this was, that if the infection level of KANZLER is low, the inoculation of the experiment failed. To detect outliers here, we defined a threshold for the mean and maximal values of the control of each experiment by using the 1.5 interquartile distance or the infected leaf area again.

The third and final quality control step was based on the variance between the biological replicates (so the same genotype was tested in two different experiments). To do so, we fitted the same model as for best linear unbiased estimation (BLUEs) and variance component estimation (see Equation 1) and defined a significant outlier threshold (p-value < 0.01) for the residuals of fitted genotypic means based on Anscombe and Tukey (1963).

All computational methods were performed within the R environment (R Core Team, 2020 version 4.0.2. using R-Studio version 1.3.1056).

Best linear unbiased estimation and variance components estimation

To estimate the effect of the design parameters and correct the phenotypic values for those, we estimated the variance components and the BLUEs of the genotypes using the phenotypic data. BLUEs of the genotypes and variance components were estimated based on the curated data. For the estimation of

variance components of the percentage of infected leaf area, we used the following linear mixed model (Hinterberger et al., 2022):

$$y = \mu + genotype + experiment + tray(experiment) + error, \quad (1)$$

where the common mean (μ) was treated as a fixed factor, whereas genotype, experiment, the tray nested within an experiment, and error effect were assumed as random factors. BLUEs were computed using the same model but assuming the genotype factor as a fixed effect. All linear mixed models were solved using the ASReml-R package Version 4 (Butler et al., 2017).

The heritability was estimated as in the following equation:

$$h^2 = \frac{\sigma_G^2}{\sigma_G^2 + \frac{\sigma_e^2}{R}}, \quad (2)$$

where $\sigma_G{}^2$ is the genotypic variance, $\sigma_e{}^2$ is the residual variance while R represents the average number of replications (independent experiments) per genotype. The standard deviation of the heritability was estimated using a bootstrapping approach by performing 500 heritability estimations using random samples that contained 80% of the total number of genotypes.

Genomic-phenomic data interoperability

In addition to the heritability as an indicator of data quality, we also assessed the genomic-phenomic data interoperability based on the genomic best linear unbiased prediction (GBLUP) for leaf infections and using publicly available GBS data (Schulthess et al., 2022).

For this prediction, we used a GBLUP model implemented in the kin.blup()-function, a wrapper for the mixed.solve()-function in the rrBLUP-Package (Endelman, 2011). The fitted mixed model can be described as follows:

$$Y = \mathbf{1}_n\mu + \boldsymbol{Z}g + e, \quad (3)$$

where Y stands for a vector of trait values for n genotypes, $\mathbf{1}_n$ is a unit vector, μ corresponds to the population mean, $\boldsymbol{Z}$ indicates a design matrix linking the elements of g to Y, g ($g \sim N(0, \sigma_g^2\mathbf{G})$) is a vector of random genotypic values and e ($e \sim N(0, \sigma_e^2\boldsymbol{I})$) accounts for the random residual term. $\mathbf{G}$ represents an additive genomic relationship matrix based on GBS marker and calculated according to the first method of VanRaden (VanRaden, 2008). $\boldsymbol{I}$ stands for an identity matrix, while σ_g^2 and σ_e^2 are the genotypic and error variance components, respectively. The assessment of the genomic-phenomic data interoperability was performed using a 5-fold cross validation approach. The "fold" means in how many subparts we split the dataset: in our case, the dataset was randomly split into five parts in each cross-validation run. In more detail, the genomic and phenotypic data of the first four parts were used as training set to predict the (fifth) remaining part (called test set) based only on the genomic data. Predictions were then compared with the observed phenotypes of the test set through correlation. The assignment of four parts to the training set and the fifth part to the test set was permutated in such a way that each subdivision served as test set only once and was four times part of the training set. The mean correlation between predicted and observed values from the five different permutations was saved for each run. We performed 500 runs of this procedure.

Data description

The here described raw data as well as BLUEs, the raw images from the detached leaf assay, and the R script to import and curate the raw phenotypic data are available in the e!DAL-PGP-Repository (Arend et al., 2014) and can be directly accessed here (https://doi.ipk-gatersleben.de/DOI/be08fbd6-4885-4f19-a849-aac73915619b/8fe440c8-6e2d-490c-841f-122bef47dfc1/2/1847940088). In more detail, the repository contains the raw images of the individual measured leaves, the raw values of the predicted infected leaf area by the open-source Python implementation of Lueck et al., 2020a, and the curated, ready-to-use data in the form of BLUEs. We also provide the images of the whole plates.

Commented [AS1]: Note: This is not the final DOI. The final DOI will be generated as soon as the review process is completed.

To comply with the FAIR principles, the data were described according to the ISA-Tab format (Sansone et al., 2012).
This includes an investigation file ("i_investigation.txt") with general information about the conditions under which the data was produced and a description of the protocols used to generate and curate the presented data. The experimental conditions and design effects of the high-throughput assay are described in the corresponding study file ("s_GB2.0_MACRO_PM.txt"). The corresponding genotype identifiers to the previously published genotypic data for the population (Schulthess et al., 2022) are also provided here. The assay file ("a_GB2.0_MACRO_PM.txt") contains the predicted infected leaf area and the corresponding image identifier for each leaf value. In addition to that, we added the minimal, mean and maximal average daily temperatures during the greenhouse period of each tested genotype to the data.
Specifically, the study file includes the effects of the experimental design of the Macrobot assay, namely the Experiments ID, the Tray ID, and the Replication Nr. Besides these, we provide the sowing, inoculation and measuring dates. The "Source Name" is the accession number from the IPK Genebank Documentation System (GBIS) combined with an internal project number reflecting the defined seed (SSD in case of PGR). Detecting mislabeling, duplicates, and correcting passport data is a well-known challenge for genebanks worldwide (Schulthess et al. 2022). For example, changes of the origin information or genotype names happen regularly. GBIS is therefore a constantly curated system and works with unique digital object identifiers (DOI) to exactly trace back requested plant material to the source accessions and their information. We include GBIS DOIs as part of the data and encourage readers and users to use them instead of genotype names to get further information and request PGR for further research and breeding activities. In addition, SAMEA (SAM, BioSample accession; E, EBI; A, Assay Sample) numbers that link phenotypes to raw sequence reads are included. Sequence data can be accessed through SAMEA numbers at https://www.ebi.ac.uk/biosamples/. The "Sample Name" is a unique identifier, connecting the genotype ID in the study-file with the raw phenotypic values in the assay file. It is also the name of the corresponding raw image.
In addition, we also provide the phenotypic data in a .csv file "raw_phenotype.csv", which is used as input by the provided R-Script. We also give access to the BLUEs for the percentage of infected leaf area based on the curated raw data. These estimates are ready-to-use for different purposes (e.g., resistance donor selection, mapping approaches, or genomic prediction).

Image data

The images generated by the Macrobot facility are the starting point for the analyses conducted. They were acquired using a Thorlabs 8050M-GE-TE camera at a resolution of 3,296 × 2,472 pixels with 365 nm (UV), 470 nm (blue), 530 nm (green), and 625 nm (red) peak wavelengths, and white light back illumination (for more details, see Lueck et al., 2020b). The raw pictures of the whole plates are saved in 16-bit TIFF format and are provided in the same repository. We cut out individual leaf positions from full plate images to allow a datapoint-wise connection of phenotypic (percentage of infected leaf area) and picture data. Those images are also provided here in PNG-format. Both sets of images have an expected resolution of 25 pixel/mm. The infected leaf area was determined on those images using the image analysis pipeline described in Lueck et al. (2020a).

Phenotypic data

The phenotypic data presented here concern the quantification of the infected leaf area. These data show the quantitative host-pathogen interaction in a controlled environment. Raw values range from 0 to 98 % infected leaf area with a mean for the whole dataset of 48.16 % (Figure 2 and 3). We observed a lower mean for the tested Elite Panel (31.87 %) and a slightly lower maximum value (94 %). In total, we measured 113,638 leaves in 422 independent experiments (Table 1) connected through the control genotype

KANZLER. On average, each genotype was tested in 1.95 experiments, with seven genotypes tested up to six times and 418 tested only once. That a genotype, besides KANZLER, was unexpectedly tested in more than two independent experiments was due to few imparities during seed logistics. In the case of genotypes tested in no more than one experiment, this was mostly due to seed availability and/or germination issues. After outlier correction, 93.4% of the raw data were considered reliable and therefore used to compute BLUEs. We excluded 3,013 datapoints (measurements of leaves) (2.7%) due to outlier correction performed based on the technical replications. Due to failed experiments, we excluded 3,827 datapoints, i.e. 3.3% of the total data collected, while 630 datapoints (0.6% of the total data) were excluded due to high differences between the biological replications.

Technical validation

We used two criteria to evaluate the data quality presented here: first, heritability, and second, cross-validated genomic prediction.
The achieved heritability of the measured host-pathogen interaction was 0.75. Variance components analysis revealed a high effect of the experimental design on the raw phenotypes. (Table 1). The performed data curation decreased the magnitude of the "Experiment" and residual effects increased in turn the variation proportion explained by the "Genotype" effect. This high heritability and the Gaussian-like distribution of the genotypic means or BLUEs (Figure 3) supports the quantitative nature of the resistance response against PM already at seedling stage.
To evaluate the genomic-phenomic data interoperability, we performed 500 runs of cross-validated genomic prediction. This analysis revealed a high prediction accuracy after data curation 0.507±0.004. In this regard, a 0.4% boost in accuracy could be attributed to the data curation steps.

Summary and outlook

We provide quantitative resistance phenotypes for 7,505 accessions of winter wheat against *Blumeria graminis*, causing PM infection at the seedling stage. Moreover, we showed that this quantification is possible and reliable using detached leaf assays – an approach traditionally used to characterize qualitative resistance. However, the method has also some of the limitations of detached leaf assays in seedlings in general:

- It is mostly well-suited for foliar diseases like leaf and stem rust, besides powdery mildew.
- The weak to moderate correlation between our high-throughput data - obtained under artificial controlled conditions with a single isolate - and field data – fully relying on natural infections (Hinterberger et al. 2022) indicates that our data should not be directly interpreted as field resistance. This most likely because natural infections are the result of a diverse population of multiple pathotypes interacting with a changing environment and the crop. We therefore presume that testing different individual isolates, which are dominant in the current pathogen gene pool, could contribute to reduce this limitation.

The assessed quantitative resistance could provide crop plant protection effects by delaying the development of the pathogen population. All in all, the here presented dataset, in combination with already available genomic information and the possibility to connect the results from this assay with other studies using the PGR population of the IPK, will serve as a good base for an educated selection. Considering the diverse origins of the phenotyped plant genetic resources (Schulthess et al. 2022) we expect to provide a valuable resource for breeders and scientists in different global regions.

Code availability

All computational methods were performed within R environment (R Core Team, 2020, version 4.0.2 using R-Studio version 1.3.1056). The code to import and curate the data ("GB2.0_Macro_PM_15.06.2022.R") is

also available at e!DAL (https://doi.ipk-gatersleben.de/DOI/dc5316a5-aad7-423b-9ce7-2d972acc0ac8/182f2ae0-6879-4f9a-980a-2c217c8e8c6b/2/1847940088).

Commented [AS2]: Note: This is not the final DOI. The final DOI will be generated as soon as the review process is completed.

Acknowledgements

The experimental work was supported by the German Federal Ministry of Education and Research within the GeneBank2.0 Project (Grant Nos. FKZ031B0184B and FKZ031B0184A) and supported by the German Plant Phenotyping Network (DPPN) (FKZ 031A053).

We thank Daniel Arend for his bioinformatic support and would like to acknowledge the following colleagues for the valuable technical help during the course of performing of experiments (in alphabetical order): Md. Al Mamum, Sonja Alner, Evangeline G. Avogadro, Federico Barbier, Ruben Betz, Gabriele Brantin, Bettina Brückner, Alessia De Matteis, Deniz Demirhan, Birgit Dubsky, André Fessel, Lena Gaczensky, Christin-Sophie Gäde, Armand Garcia, Sonja Gentz, Kathrin Gramel-Koch, Bettina Kersten, Andrea Kunze , Martina Kühne, Gabriele Lange, Ingrid Marscheider, Liana Münchhoff, Jelena Perovic, Linda Ries, Gabriele Stentzel, Julia Sturz, Jacqueline Templer, Claudia Voigt and Ellen Weiss.

We also thank Moritz Lell for his bioinformatic support and the many fruitful discussions.

Author contributions

AWS and JCR designed the study; DD generated phenotypic data; SL performed the image analysis, VH curated the data, performed quantitative genetic analyses, and wrote the manuscript with the input of all other authors.

Competing interests

The authors declare no conflict of interest.

Figure captions

Figure 1: Schematic representation of the experimental design and the workflow of the Macrobot high-throughput powdery mildew phenotyping (modified from Hinterberger et al., 2022)

Figure 2: Distribution of the raw and curated data that supports the exclusion of extreme/unexpected datapoints at levels: (1): Outlier(s) based on the technical replications of single genotypes; (2) Outlier experiment(s) based on the infection level of the susceptible control genotype; (3) Outlier(s) based on the difference in infection levels of the biological replications of single genotypes. The numbers at the top of the graph indicate the number of datapoints in each category (for details, see chapter: 'Data curation of phenotypic data')

Figure 3: Histogram of the best linear unbiased estimations of the percentage of infected leaf area of 7,505 plant genetic resources. The red dotted line represents the mean of the distribution (modified from Hinterberger et al., 2022).

Table

Table 1: Variance components and Heritability of the raw and curated phenotypic data. The factor "Experiment" refers to 446 independent experiments in which the data was generated. The Factor "Tray" refers to the tray in which the plants were grown together

	Raw Data		Curated Data	
**Component**	**Estimation**	**SE**	**Estimation**	**SE**
Experiment	198.89	14.52	157.58	11.99
Experiment:Tray	25.46	2.29	26.03	2.35
Genotype	159.77	3.73	172.69	3.92
Residual	140.16	1.89	131.26	1.83
Heritability	0.73		0.75	
SD	0.005		0.005	
Genotypes(PGR)	7,505		7,464	
Genotypes(Elite)	154		154	
Experiments	422		405	
Plates	4,887		4,694	
Lanes	14,830		14,177	
Leaves	113,638		105,647	

References:

[1] European commission. A farm to fork strategy for a fair healthy and environmentally-friendly food system; 2020; CELEX:52020DC0381.

[2] Cabrera LC, Pastor PM. The 2020 European Union report on pesticide residues in food. EFSA Journal. 2022; doi: 10.2903/j.efsa.2022.7215

[3] Lucas JA, Hawkins JN, Fraaije BA. The evolution of fungicide resistance. Adv. Appl. Microbiol. 2015; doi: 10.1016/bs.aambs.2014.09.001

[4] Calonne M, Fontaine J, Debiane D, et al. Side effects of the sterol biosynthesis inhibitor fungicide, propiconazole, on a beneficial arbuscular mycorrhizal fungus. Commun Agric Appl Biol Sci. 2011; PMID:22702206

[5] Ullah MR, Dijkstra FA. Fungicide and bactericide effects on carbon and nitrogen cycling in soils: a meta-analysis. Soil Syst. 2019; doi:10.3390/soilsystems3020023

[6] Zanin ARA, Neves DC, Teodoro LPR, et al. Reduction of pesticide application via real-time precision spraying. Sci Rep. 2022; doi:10.1038/s41598-022-09607-w

[7] McDonald BA, Linde C. The population genetics of plant pathogens and breeding strategies for durable resistance. Euphytica. 2002; doi:10.1023/A:1015678432355

[8] Wolfe MS. Trying to understand and control powdery mildew. Plant Pathol. 1984; doi:10.1111/j.1365-3059.1984.tb02868.x

[9] Torp, J. et al. Powdery mildew resistance genes in 106 Northwest European spring barley varieties. Royal Veterinary and Agricultural University Yearbook, pp. 75–102. Copenhagen, Denmark. (1978)

[10] Rubiales D, Niks RE. Characterization of Lr34, a major gene conferring nonhypersensitive resistance to wheat leaf rust. Plant Dis. 1995; doi: 10.1094/PD-79-1208

[11] Chen L. SWEET sugar transporters for phloem transport and pathogen nutrition. New Phytol. 2014; doi:10.1111/nph.12445

[12] Gupta PK. SWEET genes for disease resistance in plants. Trends Genet. 2020; doi:10.1016/j.tig.2020.08.007

[13] Niks RE., Qi XQ, Marcel TC. Quantitative resistance to biotrophic filamentous plant pathogens: concepts, misconceptions, and mechanisms. Annu Rev Phytopathol. 2015; doi:10.1146/annurev-phyto-080614-115928

[14] Hinterberger V, Douchkov D, Lueck S, et al. Mining for new sources of resistance to powdery mildew in genetic resources of winter wheat. Front Plant Sci. 2022; doi:10.3389/fpls.2022.836723

[15] Sharma S, Schulthess AW, Bassi FM, et al. Introducing beneficial alleles from plant genetic resources into the wheat germplasm. Biology. 2021; doi:10.3390/biology10100982

[16] Schulthess AW, Kale SM, Liu F, et al. Genomics-informed prebreeding unlocks the diversity in genebanks for wheat improvement. Nat Genet. 2022; doi: 10.1038/s41588-022-01189-7

[17] Lueck S, Beukert U, Douchkov D. BluVision Macro - a software for automated powdery mildew and rust disease quantification on detached leaves. J Open Source Softw. 2020a; doi:10.21105/joss.02259

[18] Lueck S, Strickert M, Lorbeer M, et al. "Macrobot": an automated segmentation-based system for powdery mildew disease quantification. Plant Phenomics. 2020b; doi:10.34133/2020/5839856

[19] R Core Team. R: A language and environment for statistical computing. R Foundation for Statistical Computing, Vienna, Austria. 2020; URL: https://www.R-project.org/

[20] Anscombe FJ, Tukey JW. The examination and analysis of residuals. Technometrics. 1963; doi:10.2307/1266059

[21] Butler DG, Cullis BR, Gilmour AR, et al. ASReml-R reference manual version 4. VSN International Ltd, Hemel Hempstead, HP1 1ES, UK. 2017; URL: https://asreml.kb.vsni.co.uk/wp-content/uploads/sites/3/ASReml-R-Reference-Manual-4.pdf.

[22] Endelman JB. Ridge regression and other kernels for genomic selection with R package rrBLUP. Plant Genome. 2011; doi:10.3835/plantgenome2011.08.0024

[23] VanRaden PM. Efficient methods to compute genomic predictions. J Dairy Sci. 2008; doi:10.3168/jds.2007-0980.

[24] Arend D, Lange M, Chen J, et al. e!DAL - a framework to store, share and publish research data. BMC Bioinformatics. 2014; doi:10.1186/1471-2105-15-214

[25] Sansone SA, Rocca-Serra P, Field D, et al. Toward interoperable bioscience data. Nat Genet. 2012; doi:10.1038/ng.1054

Figure 1

Click here to access/download;Figure;Figure 1 PM2_revised.pdf

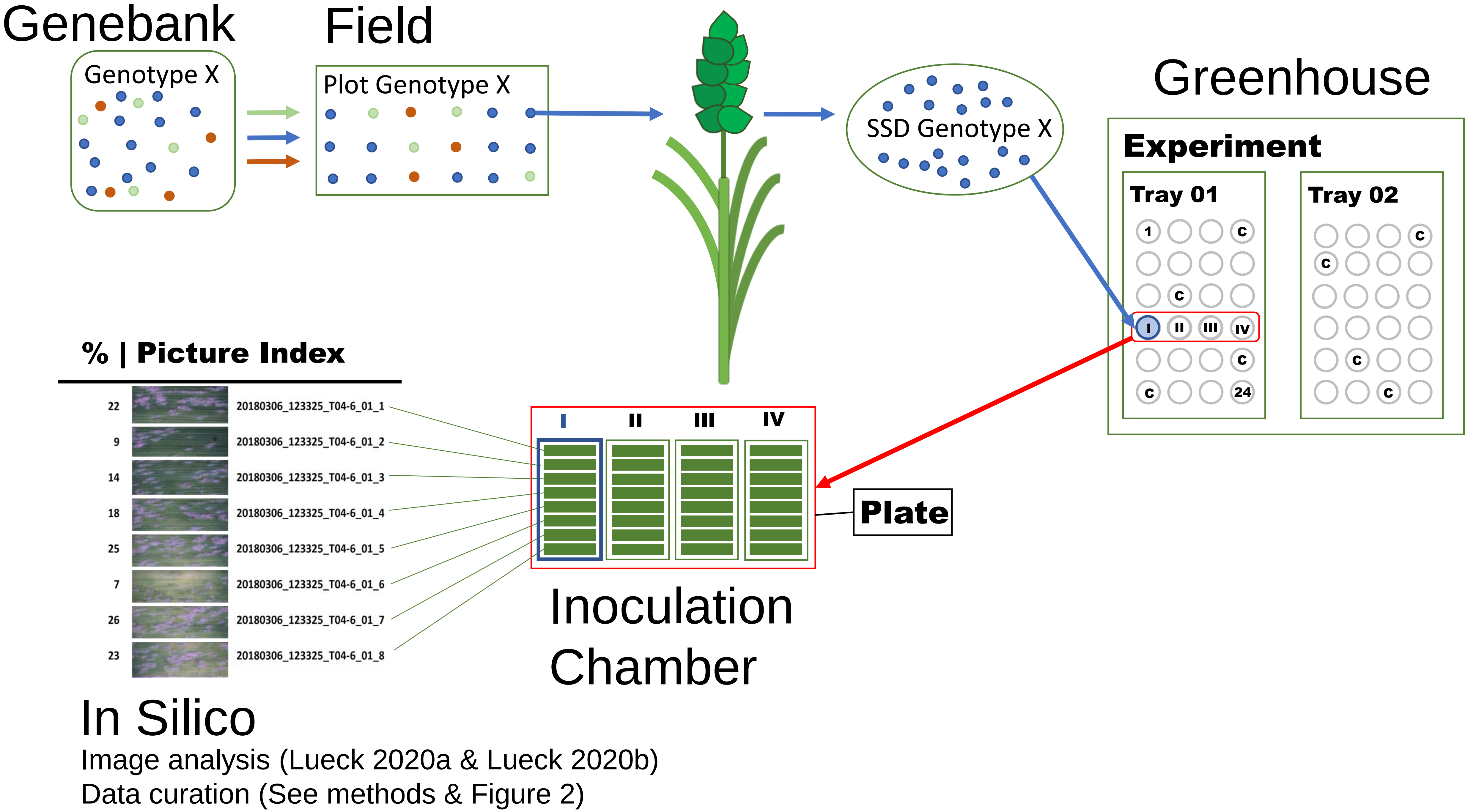

Figure 2

Click here to access/download;Figure;Figure 2 PM2_revised.pdf

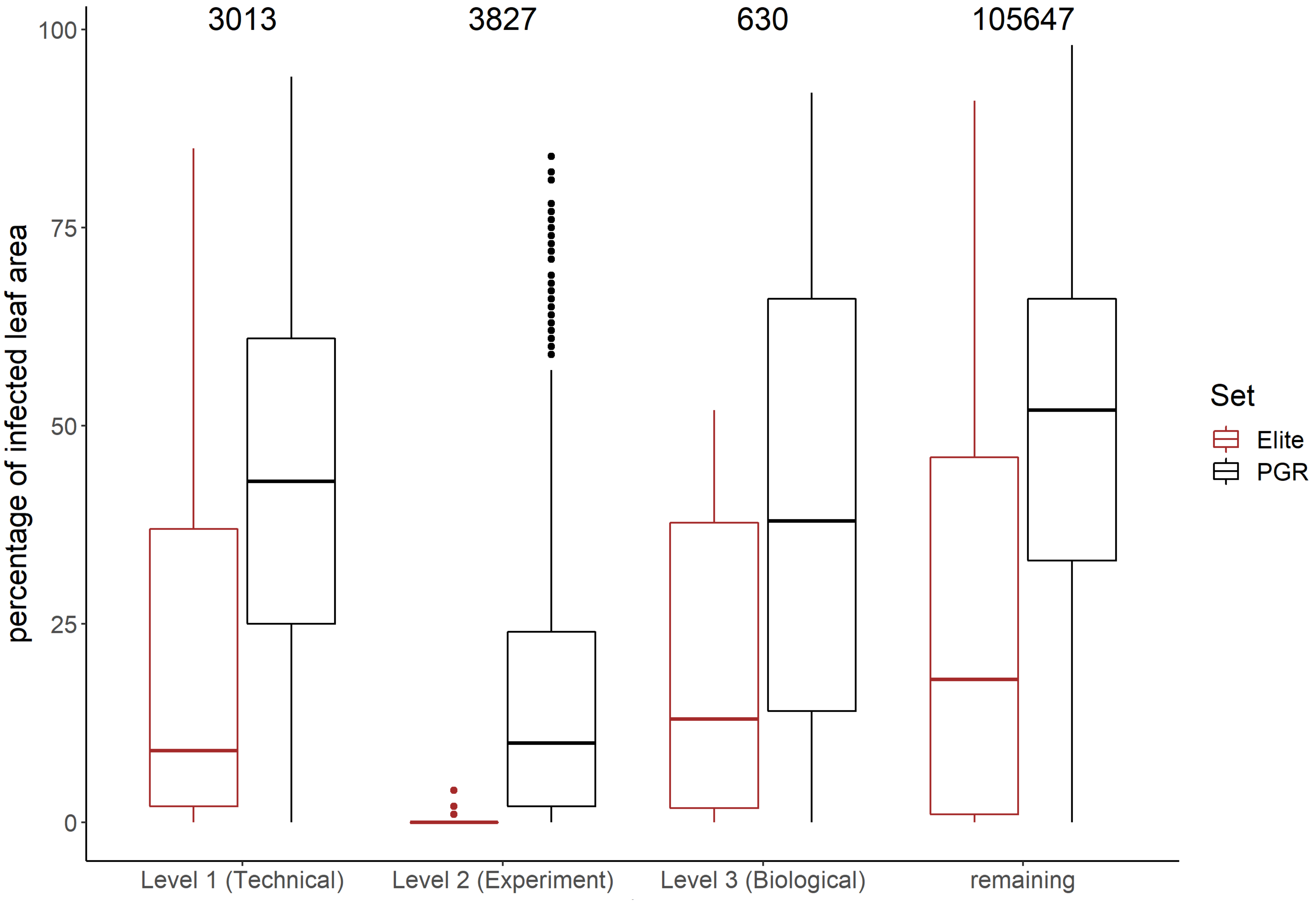

Figure 3

Click here to access/download;Figure;Figure 3 PM2_revised.pdf

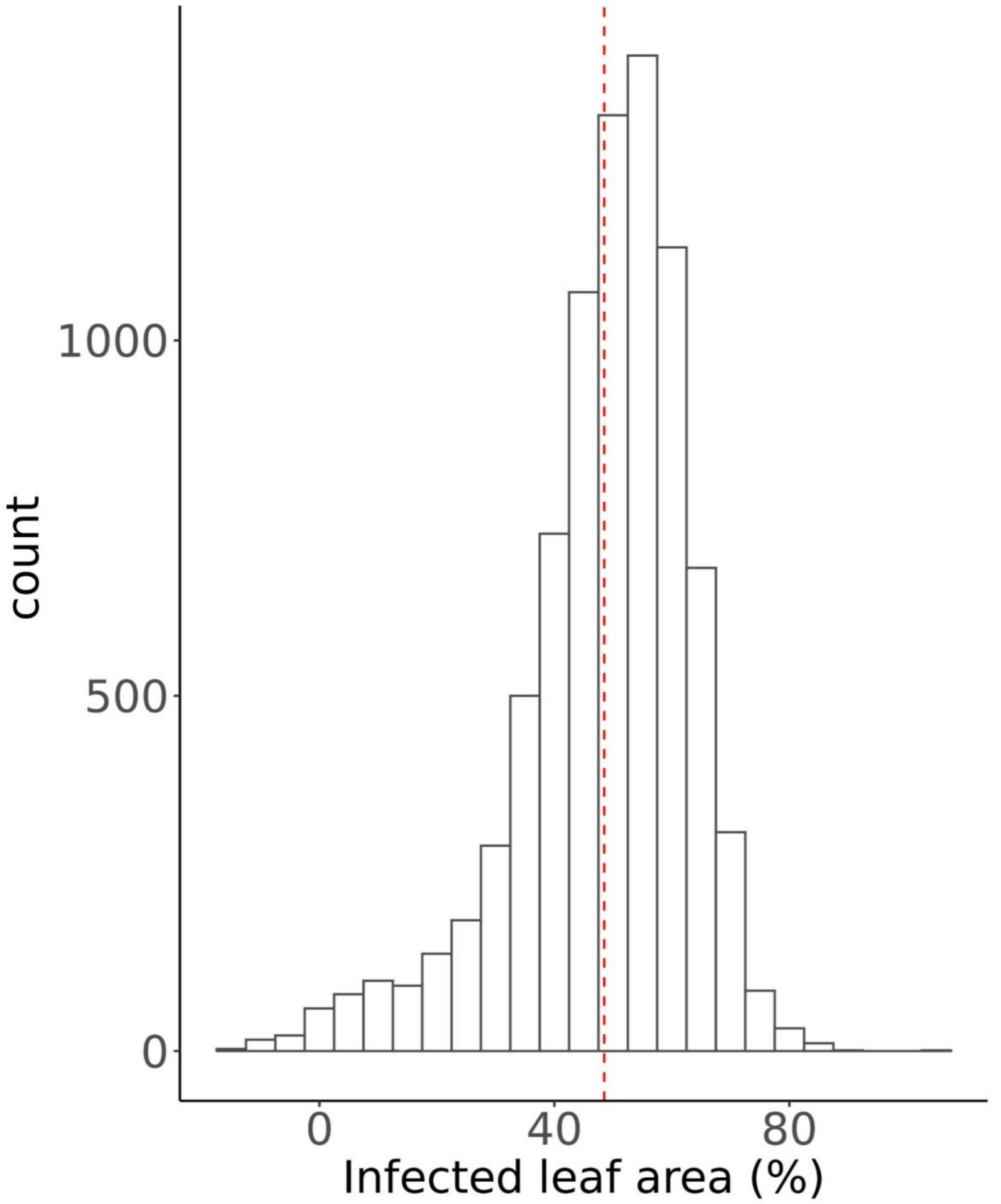

Supplementary Table 1

Click here to access/download
Supplementary Material
Supplement_TableS1 (Pathotype pattern of FAL 92315).xlsx

Click here to access/download;Personal Cover;Cover_letter.docx

Dear Hans,

We thank you and the four reviewers for the very constructive feedback regarding our dataset and the *Data Note* manuscript entitled "High throughput imaging of powdery mildew resistance of the winter wheat collection hosted at the German Federal ex situ Genebank for Agricultural and Horticultural Crops" - written by Valentin Hinterberger and colleagues. These suggestions and remarks helped us to improve the quality of the manuscript as well as the presentation and accessibility to the data themselves. In this letter, we include a detailed assessment and response to all points raised by the reviewers. Each comment is followed by its corresponding response, which is also indicated with the bold capital letter "**R**". Changes performed in the manuscript can be checked using the "Tracking Changes" functionality of *Microsoft Word*. To facilitate the review process, the text positions of these changes are also indicated between brackets in the present letter based on the "Simple Markup" mode. We hope that the revised version of our manuscript and dataset now fulfills the publication requirements of *GigaScience*.

Many thanks in advance for your time and interest

Best regards,
Albert W. Schulthess

Reviewer 1: Antonín Dreiseitl

General comment: My recommendation: Reject
In the introduction of my critical review I should say that I am plant pathologist who have studied disease resistance of many thousands the World gene banks accessions. In spite of my long-term experience I didn´t find a sense of the job presented in the manuscript. I am omitting general issues in my introduction. My comments are marked with an asterisk (*).
R:
Dear Antonín Dreiseitl,

Many thanks for your very detailed review. Your constructive criticism substantially contributed to broaden the discussion about the advantages and limitations of our methods and data, especially regarding qualitative-vs-quantitative resistance. When we perceived that some of your critical points were somehow related, we decided to merge them together and provide also an integrated answer to them. We hope that this way of presenting your points and our answers make this letter less redundant and easier to read.

Best regards,
Albert W. Schulthess

Comments on data accessibility and value: Main reservations. Genebanks worldwide are transforming into bio-digital resource centres, providing not only access to the plant material itself but also to its phenotypic and genotypic information. … Here we provide phenotypic data for the resistance against Blumeria graminis. * Due to technical obstacles and no time and taste to solve them I didn´t see any your pictures (for all readers including reviewers pictures must be easily seen!). "This massive amount of phenotypic data combined with the already published genotypic data also provides a valuable and unique training dataset for the development of novel genotype-based predictions as well as mapping methods". * I do not see any value of the set of described pictures.
R:
We are very sorry that you were not able to access the images stored in the data repository. We reuploaded the data into e!DAL, which can be now accessed by following this link: https://doi.ipk-gatersleben.de/DOI/be08fbd6-4885-4f19-a849-aac73915619b/8fe440c8-6e2d-490c-841f-122bef47dfc1/2/1847940088. Following the remarks from reviewer #4, we included the original 16-bit TIFF images of whole plates in addition to the images of the separated leaves as part of the repository. This is now indicated in our revised manuscript (please read page 6, lines 232 to 234). We expect that all these changes highlight the value of the provided images.

Comments on qualitative vs quantitative resistance in seedlings: But according of the text authors consider phenotype as quantitative parameter - severity of the disease on leaves in juvenile stage of the host! (on second leaves of 14 days old plants). Disease resistance phenotype can be characterise only describing of the resistance response (RT) to an isolate - qualitative development of the pathogen on the host genotype. For that usually 0 to 4 scale (or 1 to 9 –including intertypes) is used showing e.g. RT1 = mycelium of the pathogen is seen but conidia are not developed. Other, usually less important characteristics can be added (e.g. occurrence of chlorosis or necrosis – spots). Based on the gene-for-gene model and in the given stage of the host you could see only if the isolate is virulent or avirulent to a gene bank accession

(if an accession is or isn´t resistant to one isolate). Nothing else. You quantified RTs but it has no importance at all. *Conclusion:* You didn´t provide phenotypic data for the resistance against Blumeria graminis. "We quantified the resistance reaction (of the gene bank accessions)"… * You didn´t quantified the resistance reaction, you quantified severity of the isolate developed on the host genotypes. Resistance in the given case means qualitative response of the pathogen on the host. The phenotypic data presented here concern the quantification of the infected leaf area. * Quantification of the infected leaf area in juvenile stage can´t be consider as phenotypic data for the disease resistance. These data show the quantitative host-pathogen interaction in a controlled Environment. * Quantitative aspect has no importance in juvenile stage of the host. Since the detached leaf assays are a standard method in phytopathology, cheap, easy, and repeatable quantification methods are highly desirable. * I do not see any contribution of quantification methods for qualitative disease resistance.

R:

Thanks for rising all these points. As you mention, detached-leaf assay evaluations are generally performed in a qualitative (usually manually) manner and focusing on mycelium growth, sporulation as well as the development of chlorosis/necrosis (Torp et al., 1978, for instance). We agree with you as well that the gene-for-gene model presents the host-pathotype interaction as qualitative due to the detection or not detection of elicitor molecules by the host and the resulting 0|1 (binomial) pattern of the hypersensitive reaction (Niks et al., 2015). However, there is evidence for quantitative resistance mechanisms in seedlings, thus implying that seedling resistance is not necessarily of qualitative nature. For example, *Lr34* (Rubiales and Niks, 1995) confers partial resistance already in seedling stage and encodes for an ABC transporter (Krattinger et al., 2009). There are also genes like the *SWEET* genes which confer quantitative susceptibility (Chen et al., 2014 Gupta, 2020) and are also expressed in seedling stage. Many effects of quantitative resistance mechanisms are more pronounced in the field context and with adult plants, like latency effects for example. This is the reason why quantitative and adult plant resistance or field resistance are often used synonymously (Niks et al., 2015). A plant could be qualitatively susceptible (can be infected by the pathogen), but quantitatively resistance by delaying the development of the pathogen. This latency mechanism has positive effects for disease and resistance management at the small (crop) and large (agroecological) scale, due to reduced spore production. We therefore aimed to measure this latency effect in our setup, where the environment conditions are optimal and highly controlled for pathogen development. In this context, the high heritability (0.75) estimated for our resistance quantification at the seedling stage as well as the Gaussian-like distribution of the genotypic means (Figure R1, Hinterberger et al., 2022) are indicators of a quantitative genetic basis and supports our hypothesis. We discussed these points in the revised manuscript (please read page 3, lines 64 to 77; page 7, lines 256 to 261).

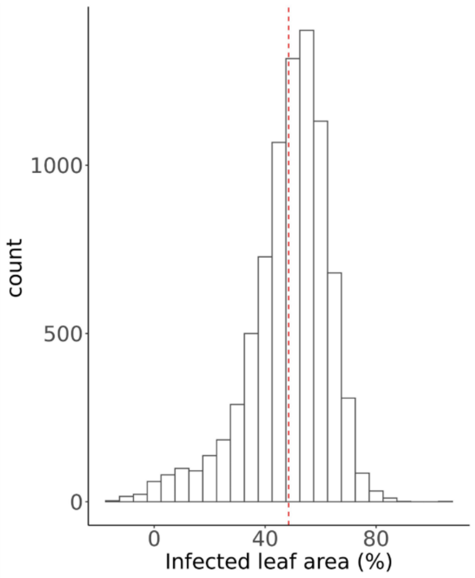

Figure R1. Distribution of genotypic means of the percentage of infected leaf area of 7,505 plant genetic resources. The dashed red line indicates the mean value (modified from Hinterberger et al., 2022).

Comments on resistance breeding and its long-term field management: Increasing resistance diversity is one way to stabilize yields without fungicides. * This is a concept that has long since been surpassed at least for mildew on cereals (e.g. McDonald and Linde 2002). As a component of genome-wide mapping approaches, this data is a valuable source of information on donors for potentially novel resistance genes, as we recently have shown (Hinterberger et al., 2022). * There is much shorter way for this purpose; you can detect specific resistance genes only. Using of such genes is a blind way for protection of the crop against the disease.

R:

Many thanks for these comments. According to McDonald and Linde (2002), resistance breeding based on broadly deployed qualitative resistance QTL is affected by “Boom and Bust” cycles, due to the high selection pressure observed in highly uniform host populations. This makes major QTL resistance a less sustainable strategy for plant protection in the long term. McDonald and Linde (2002) proposed in contrast that diversifying resistance mechanisms through quantitative resistance, cultivar mixtures or multilines is a more sustainable way to manage resistance against *Blumeria graminis* in the long term when plant protection fully relies on the use of resistant cultivars (Figure R2). In this context, an increase of diversity regarding resistance mechanisms in the breeding pool should delay the rise of pathogen epidemics and stabilize the resistances in the long term. Therefore, we expect that our quantitative resistance data contribute to the future discovery of basal resistance mechanisms that provide more durable resistance. We mentioned these points throughout the revised manuscript (please read page 3, lines 64 to 77; page 7, lines 256 to 261; page 7, lines 279 to 280).

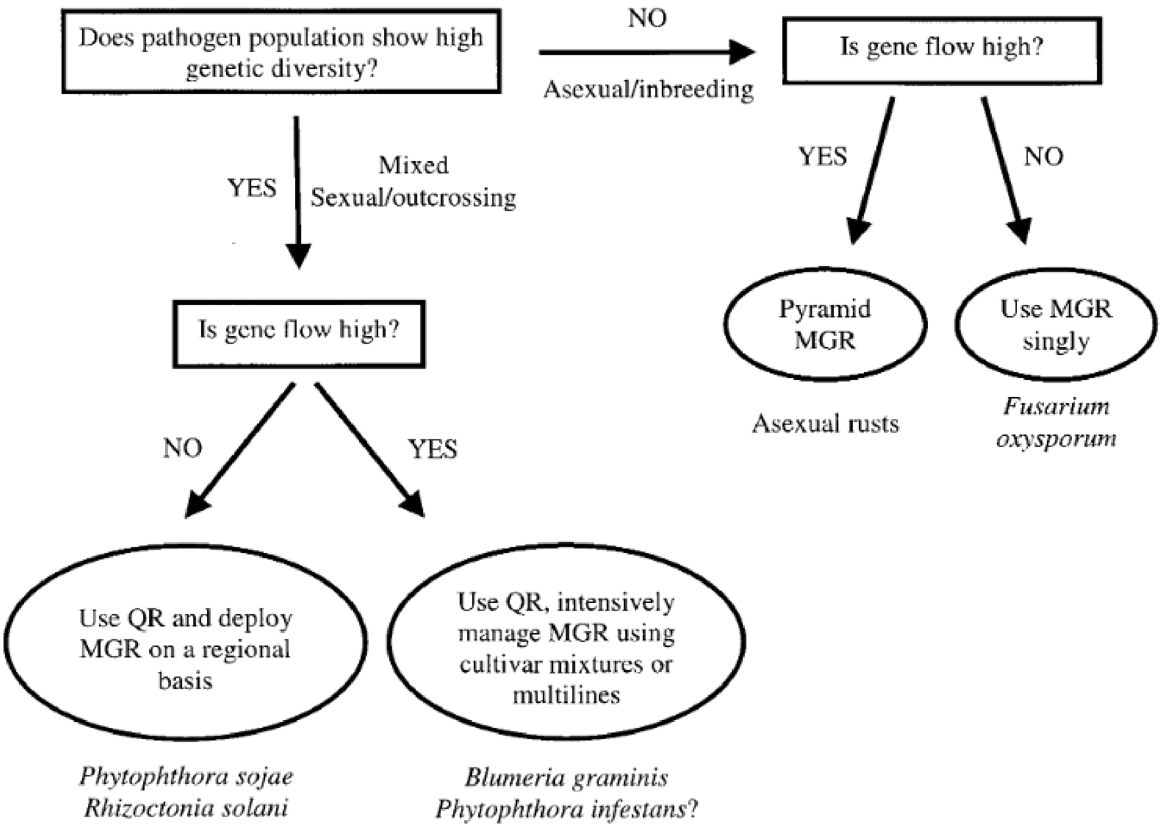

Figure R2. A simplified decision diagram to assist with developing resistance-breeding strategies to achieve durable disease resistance. MGR = Major Gene Resistance, resistance that has large effects, is based on the hypersensitive response and follows the receptor-elicitor model of the gene-for-gene interaction. QR = Quantitative Resistance, resistance that has small, nearly equal, and additive effects that are equally effective against all strains of the pathogen (Original figure from McDonald and Linde, 2002).

Comment on mislabeling of genebank accessions: * You didn´t verify authenticity of the accessions and in spite of it you would like to create patterns of them for the future. However, mislabeling occur in high percentage of accessions in all gene banks including developed countries.

R:

Many thanks for mentioning this non-trivial issue that occurs in genebanks. The IPK genebank is constantly curating the taxonomical identity of accessions. If changes in descriptors of accessions are made/identified, the genebank information system (GBIS) correspondingly updates their databases. In this respect, passport data of accessions are dynamic in time. To deal with different versions of passport data, GBIS generates unique digital object identifiers (DOIs) that do not change overtime and can exactly track changes in the passport information of each accession – this including accession numbers themselves. This is the reason why we explicitly encourage readers to access plant material (their information and seeds) using the provided genebank DOIs. Moreover, the dynamic nature of identifiers and passport data from genebanks might not be well known for readers in general. Therefore, we briefly commented on this issue in the revised manuscript to warn readers and data users when they intend to access IPK genebank material (please read page 6, lines 213 to 219).

Comment on heterogeneity within genebank accessions: We used a "single seed descent" step to reduce genetic heterogeneity within accessions by sampling one genotype per homogenous accessions and two genotypes if accessions were heterogenous. * How did you find „heterogeneous“ accessions and what was their percentage? Did you know how many genotypes are present in a heterogeneous accession? With using of one isolate you could find heterogeneity in low number of accessions and at maximum two genotypes.

R:

Many thanks for this comment. Heterogeneity of genebank accessions was assessed during field propagation and fully based on morphological traits such as spike form, plant height, color and architecture. In more detail, among 7,505 accessions for which SSD-seeds were obtained, only three were clearly heterogenous. In this respect, we were able to identify two main plant types per heterogenous accession and treated them as two separate genotypes during the whole process. We briefly report this now in the revised manuscript (please read page 3, lines 97-99).

Comment on pathogen isolate: * You used one isolate only. According of you it was „highly aggressive“ and „highly virulent“ – I do not know what means „highly virulent“, probably an isolate with a broad spectrum of virulences. But even this one isolate is not characterised. Without knowing of its full virulence spectrum to all known resistance genes the value of results is close to zero.

R:

Many thanks for highlighting this point. A detailed characterization showing the broad virulence spectrum of the used isolate (FAL 92315) is included in the Supplementary Table 1 of our revised manuscript.

Comment on additional environment data: * Your test were conducted in „controlled environment“, however no information about it during growing of accessions before inoculation. Light intensity during incubation is also missing.

R:

Many thanks for this remark. As also suggested by reviewer #2, we added the minimal, mean and maximal average daily temperatures during the greenhouse period of each tested sample to the data. This is now mentioned in the manuscript (please read page 6, lines 206 to 208) and you can also access to this information by following this preliminary link at e!DAL: https://doi.ipk-gatersleben.de/DOI/be08fbd6-4885-4f19-a849-aac73915619b/8fe440c8-6e2d-490c-841f-122bef47dfc1/2/1847940088. Furthermore, artificial light intensity parameters (16 h light, 15 $\mu E\ m^{-2}\ s^{-1}$) are now included in the revised manuscript (please read page 4, line 119)

Reviewer #2: Feng Hui

Comment #1: This paper addresses the problem of resistance traits and provides phenotypic data on resistance to powdery mildew, providing a dataset for the development of genotype-based prediction and mapping methods.
R:
Many thanks for positively highlighting the main features of our data.

Comment #2: The resolution of Fig1 and Fig2 is too low.
R:
Thanks for noticing this issue. The resolution of both images is improved in the revised version of the manuscript.

Comment #3: The result and summary part should be added.
R: Thanks for this suggestion. In consultation with the editor and in accordance with the guidelines of *GigaScience* for data notes, a results section is not needed. However, we have added a "Summary and outlook" section (please read page 7, lines 265 to 284).

Comment #4: The article does not cite studies similar to the topic for comparison. What are the advantages and limitations of this topic for previous studies?
R:
Many thanks for touching this point. Detached leaf assays of the wheat-*Blumeria graminis* pathosystem have been widely used in the past. However, original phenotypic data is usually not available in those publications or, in the best case, only manually derived qualitative phenotypes are presented (commonly using a 0-4 scale like in Torp et al., 1978). Furthermore, in previous studies the isolates used for infection differ and their virulence spectra are in some cases not well described, which limits comparisons with other studies. In addition, past studies are not high-throughput and, to the best of our knowledge, cover only a limited number (hundred to thousand) genotypes (Parks et al., 2008, Liu et al., 2000, Xie et al., 2020, Li et al., 2019). The weak correlation between our high-throughput data obtained under artificial controlled conditions and field data relying on natural infections (Hinterberger et al. 2022) indicates that our data should not be directly interpreted as field resistance. This most likely because natural infections are the result of a diverse population of multiple pathotypes interacting with a changing environment and the crop. Nevertheless, detached leaf assays offer a much less chaotic system and the opportunity to investigate in a more precise fashion the biological interaction between plants and a well-defined pathogen. Several of these points are also mentioned in the manuscript (please read page 3, lines 64 to 77; page 7, lines 256 to 261; page 7, lines 265 to 284) while the advantages of the data publication itself are mentioned in the answer to your comment #13.

Comment #5: The weather conditions during planting should be added.
R:
Many thanks for this comment. Besides the controlled moisture, temperature and artificial light conditions during infection, which can be found in the revised manuscript (please read page 4, lines 118 to 119), we added the minimal, mean and maximal average daily temperatures during the plant growing period under greenhouse conditions for each sample as part of the available data (https://doi.ipk-gatersleben.de/DOI/be08fbd6-4885-4f19-a849-aac73915619b/8fe440c8-

6e2d-490c-841f-122bef47dfc1/2/1847940088). This added information is now also reported in the revised manuscript (please read page 6, lines 206 to 208).

Comment #6: How does the sampling area and sampling time affect the experimental results?
R:
This is an interesting question. Our sampling procedure considered only the middle part of the second leaf (two-leaf stage), because early trials evidenced that the base of the leaf is more susceptible to powdery mildew, while the tip is more resistant (data not shown). This is now briefly mentioned in the manuscript (please read page 3, line 108 to page 4, line 110). Since plants were tested during a very early developmental stage, we were not able to investigate the effect of the sampled area size on the infection reaction. However, the minimum number of three leaves per genotype is a representative sample size and provided thus enough leaf surface to observe clear symptoms in the susceptible control genotype: KANZLER. Moreover, seedling and adult plant resistances are impaired due to morphological and physiological changes related to plant defense as well as differential expression of resistance genes during plant development (Niks et al. 2015). Nevertheless, we anticipate from our leaf rust experiments (Beuker et al. 2021) that investigating such effects is challenging for the experimental setup of our high-throughput phenotyping platform. This because maintaining plants completely free of diseases becomes very difficult during later developmental stages under greenhouse conditions without the help of fungicides. In this sense, both greenhouse pathogens and fungicides would add statistical noise into the system. Although we cannot confirm that developmental effects on powdery mildew resistance can be captured by our high-throughput phenotyping platform, these effects can be discarded from our conducted experiments, because the sampling time was fixed to a specific developmental stage for all tested plant material: the second leaf of 14-day-old seedlings.

Comment #7: The overall flow chart of experiment should be added.
R:
Many thanks for this suggestion. We extended Figure 1 for this purpose.

Comment #8: Line 286, "some tested up to 6 times and others tested only once". Why did this happen?
R:
Many thanks for this remark. We are very sorry that the former sentence was not specific enough regarding this point, giving thus the false impression that fluctuations in the number of testing instances for single genotypes were a generalized problem. Due to a limited number of available seeds, insufficient germination capacity and limited early plant development, 418 genotypes could be tested only once. Moreover, only seven genotypes were tested more than twice (Table R1), which is a very marginal proportion considering the total number of tested genotypes.

Table R1. Genotypes tested more than twice

Genotype	Times tested
Benchmark_6108	6
Bussard_6118	4
Cellule_6120	4

Dekan_6126	4
Ponticus_6208	4
Tobak_6233	4
TRI_16612_7931	3

All genotypes names were coded during seed handling, experiment preparation and testing. In this sense, their exact identities (genotype names) were mapped afterwards to generate the metadata information. In this context, the very low proportion of plant material tested with more than two replications were most likely due to imparities during seed logistics that could not be early detected because the handled material was coded. These details are now also clearly specified in the manuscript (please read page 7, lines 244 to 248).

Comment #9: Line 287, What are the criteria for outlier correction?
R:
Thanks for rising this question. Three levels of outlier correction with different cutoff criteria were considered: at the level of technical replications, control genotypes as well as statistical (biological) replications. This is described in detail in the “Data Curation” section (Methods) of the revised manuscript (please read page 4, lines 134 to 147).

Comment #10: What is the fold of the cross verification? What is the basis for grouping?
R:
Thanks for this comment. We are very sorry that we were not clear enough in the former version of our manuscript regarding cross-validations. The “fold” means in how many subparts we split the dataset: in our case, the dataset was randomly split into five parts in each cross-validation run. In more detail, the genomic and phenotypic data of the first four parts were used as training set to predict the (fifth) remaining part (called test set) based only on the genomic data. Predictions were then compared with the observed phenotypes of the test set through correlation. The assignment of four parts to the training set and the fifth part to the test set was permutated in such a way that each subdivision served as test set only once and was four times part of the training set. The process of subdividing the dataset into five different parts was repeated 500 times. These details can be found now in the “Genomic-phenomic data interoperability” section of the revised manuscript (please read page 5, lines 180 to 189).

Comment #11: How much phenotypic data were extracted in this paper?
R:
Many thanks for pointing this out. After our multiple-level data curation procedure, 6.6 % of the total datapoints were discarded from the original dataset. Moreover, Figure 2 summarizes how much data was still available after each data curation level. Further details can be found in the revised manuscript (please read page 7, lines 248 to 252).

Comment #12: How to do the "Genomic-phenomic data interoperability"? What is the result? It is better to attach the operation process and video.
R:
Thanks for this question. "Genomic-phenomic data interoperability" was tested by performing 5-fold cross-validation. If genomic prediction is accurate, there is a good connection between the phenotypic and genotypic data. More details can be found in our response to your comment

#10 as well as in the chapter "Technical Validation" of the manuscript (please read page 7, lines 262 to 264).

Comment #13: Each part of the content has been published separately. What is the innovation of this paper?

R:

Many thanks for rising this point. The raw phenotypic data (percentage of infected leaf area) and their basis (processed and raw pictures) have not been published so far. The nature and size of this dataset is therefore unique, and its being provided by following the FAIR principles of data publication. Furthermore, the dataset is made available under a CC0 license, allowing thus its reuse for scientific as well as commercial purposes. Moreover, we also provide connectivity to genomic data published elsewhere (Schulthess et al. 2022), which will allow further applications. Follow up experiments can be performed as well by requesting seeds from the German genbank using the provided DOIs. All these points are being highlighted in different sections of the revised manuscript (please read page 3, lines 78 to 85; page 5 lines 168 to 170; page 6, lines 198 to 237).

Reviewer #3: Rohit Mago

Comment #1: The manuscript provides phenotypic data for the resistance against powdery mildew on leaves of 7398 winter wheat accessions held at German Federal Ex Situ Gene Bank for Agricultural and an additional 150 commercial cultivars. The authors used a high-throughput system for phenotyping and provide over 110,000 pictures at high resolution of these genotypes infected with powdery mildew race FAL 92315. The data will be useful for other researchers and breeders to pick resistant germplasm in their breeding programs and the phenotypic data set can be used for AI based training for novel genotype-based predictions as well as mapping methods. The manuscript is well written and warrants publication.
R:
We are very grateful for this positive feedback.

Comment #2: However, there are a few questions that authors should address in the manuscript including limitations of the technique. The authors used only a single virulent powdery mildew race in their experiments thus creating a bias. While the results will be useful for breeders to use the genotypes in Germany, they may not be useful for breeders in other locations due to prevalence of other pathotypes
R:
Many thanks for this remark. Given the information formerly provided by us and the fact that the global pathotype population of *Blumeria graminis* is very diverse and fast evolving, we can understand very well your remarks. Nonetheless, the used isolate FAL92315 is highly virulent (please see the newly added Supplementary Table 1 and page 8, lines 283 to 284 of the revised manuscript). Due to the known virulence pattern, it is possible to connect the data also with experiments with other pathotypes with known virulence spectrum. Moreover, considering the distribution of FAL92315 infections according to the diverse origins of the phenotyped plant genetic resources (Figure R3), we did not observe any obvious population structure associated with infections. Therefore, we expect to provide a valuable resource for breeders in different global regions.

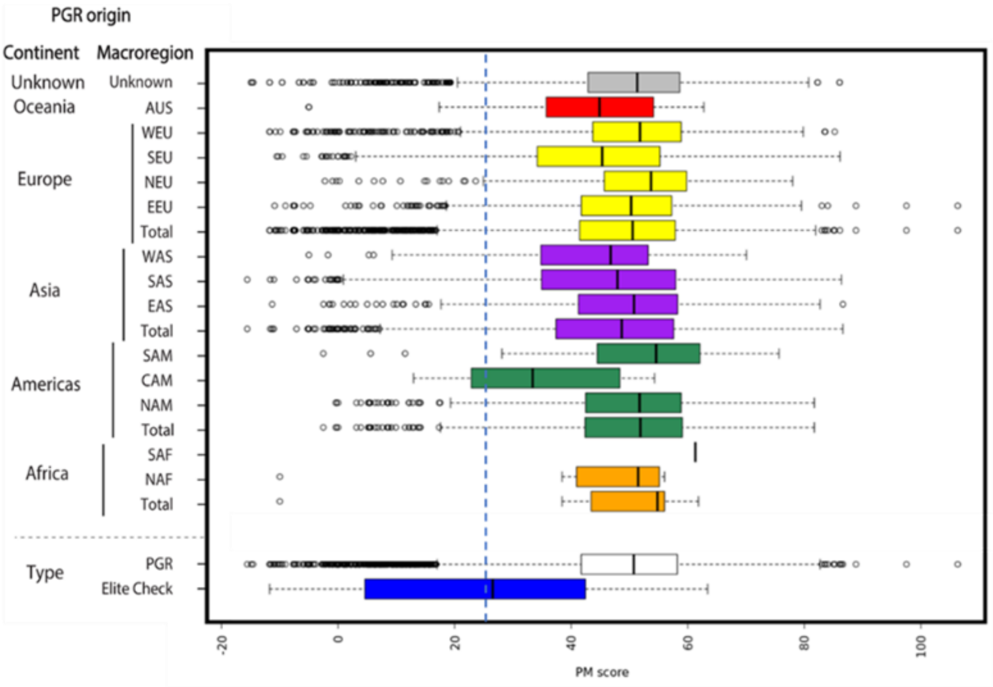

Figure R3. Best linear unbiased estimations (BLUEs) for 7,464 plant genetic resources (PGR) of wheat stored at the German Federal ex situ Genebank for Agricultural and Horticultural Crop Species (IPK-genebank) along with 154 European elite cultivar checks screened for resistance against powdery mildew (PM, *Blumeria Graminis*) using detached leaf assays and the isolate FAL92315. Resistance is quantified in percentage of infected leaf area corrected by the inoculation group effect. BLUEs that lie outside of the 0-100 parametric space are due to the unorthogonal data structure of experiments. According to their origins, PGR can be classified into five different continents, namely Africa, Americas, Asia, Europe and Oceania, or their origins are unknown. Continents are further subdivided into 13 megaregions: Northern (NAF) and South (SAF) Africa, Central (CAM), Northern (NAM) and South (SAM) America, Eastern (EAS), Southern (SAS) and Western (WAS) Asia, Eastern (EEU), Northern (NEU), Southern (SEU) and Western (WEU) Europe, as well as Australia and New Zealand (AUS). Boxes enclose 50% of the central data, including median (vertical black bold line), while whiskers are ± 1.5 × interquartile range and dots represent extreme values. The vertical blue dashed line corresponds to the average of check cultivars.

Comment #3: The high-throughput phenotyping may not be suitable for diseases where it is not possible to perform detached leaf assays like powdery mildew.

R:

Many thanks for highlighting this. We fully agree with you that detached leaf assays are in general best suited for foliar diseases like leaf rust and yellow rust, besides powdery mildew. We briefly commented on this point in the manuscript (please read page 7, line 271).

Reviewer #4: Chris Armit

Comment: This Data Note describes a high-throughput imaging approach for evaluating of powdery mildew resistance in wheat. I was invited to check the quality of the image data, and this review reports on the image data aspect of the manuscript. In the manuscript, the authors state the following: "The images generated by the Macrobot facility are the starting point for the analyses conducted. They were acquired using a Thorlabs 8050M-GE-TE camera at a resolution of 3,296 Å~ 2,472px with 365 nm (UV), 470 nm (blue), 530 nm (green), and 625 nm (red) peak wavelengths, and white light back illumination (for more details, see Lueck et al., 2020b). The raw pictures of the whole plates are saved in 16-bit TIFF format. We separated the images of individual leaves to allow a datapoint-wise connection of phenotypic data and picture data. Those images are provided here in PNG-format." From this description, I was anticipating a high-resolution, multi-spectral imaging dataset of sufficient quality for plant phenomic image analysis. However, on downloading the image data from e!dal, I have observed that the PNG image data is of low-resolution, and extremely grainy. To illustrate with one example, the PNG image [20191029_122731_exp56_P12-6_02_7.png] in directory [exp56_6] is only 179 x 58 pixels. In the manuscript, the authors further state the following: "The infected leaf area was predicted on those images using the image analysis pipeline described in Lueck et al. 2020a." This sentence is ambiguous and I am unsure as to whether the phenomic analysis was performed on the high-resolution TIFF images, or the low-resolution PNG images. Consequently, I have two questions for the authors relating to this manuscript and the accompanying dataset. Can the authors please confirm whether the image analysis outlined in the manuscript was performed on high-resolution TIFF images? Alternatively, was the image analysis performed on low-resolution PNG images? Will the original 16-bit TIFF images of whole plates be made publicly available in support of this study?

R:

Many thanks for this very helpful remark. As presented in Figure R4, the row - and leaf recognitions were performed on the full-size TIFF images of the entire plate (3296 x 2472 pixels). After this, cutouts of the individual leaves were stored in PNG format. The analysis of infected area was done on the PNG leaf cutouts. Both full-size TIFFs and leaf-PNG cutouts have the same image resolution of 25 pixels/mm. This is now also well specified in the manuscript (please read page 6, lines 233 to 237). Moreover, we added the original 16-bit TIFF images of the whole plates to the repository in addition to the images of the separated leaves. Please check the following preliminary link at e!DAL:https://doi.ipk-gatersleben.de/DOI/be08fbd6-4885-4f19-a849aac73915619b/8fe440c8-6e2d-490c-841f-122bef47dfc1/2/1847940088.

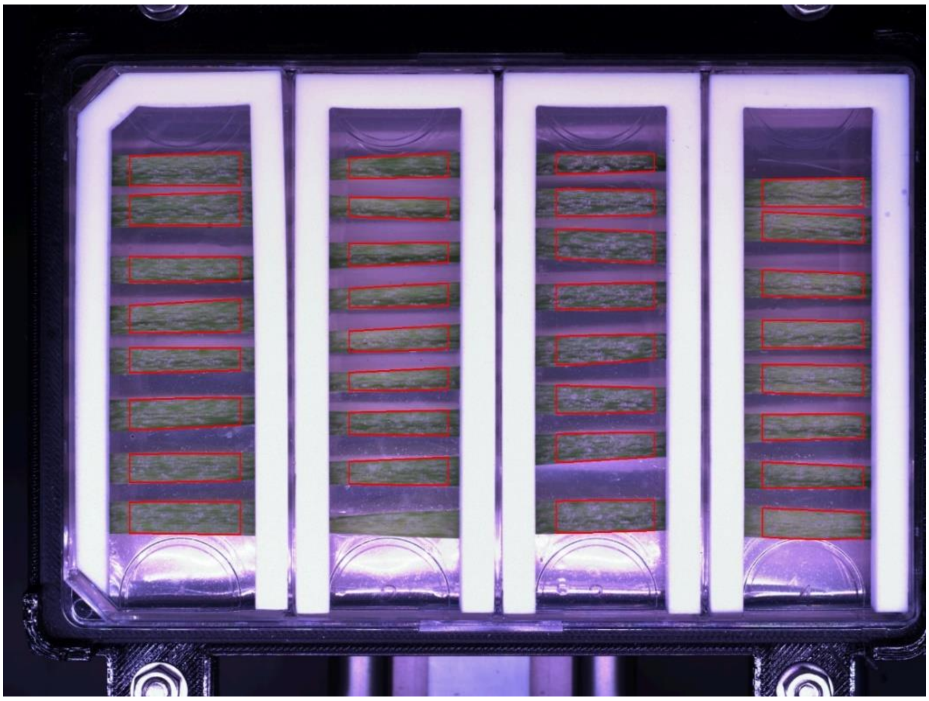

Figure R4. Picture of an entire inoculated plate. Recognized leaf segments are delineated with red segments.

Cited literature in responses of the authors

- Beukert, U. et al. Efficiency of a seedling phenotyping strategy to support European wheat breeding focusing on leaf rust resistance. Biology, (2021). doi: 10.3390/biology10070628.
- Chen, L. SWEET sugar transporters for phloem transport and pathogen nutrition. New Phytologist (2014). doi:10.1111/nph.12445
- Gupta, PK. SWEET genes for disease resistance in plants. Trends in Genetics (2020). doi:10.1016/j.tig.2020.08.007
- Hinterberger, V. et al. Mining for new sources of resistance to powdery mildew in genetic resources of winter wheat. Front Plant Sci, (2022). doi: 10.3389/fpls.2022.836723.
- Krattinger, SG. et al. A putative ABC transporter confers durable resistance to multiple fungal pathogens in wheat. Science, (2009). doi: 10.1126/science.1166453
- McDonald, B.A., Linde, C. The population genetics of plant pathogens and breeding strategies for durable resistance. Euphytica (2002). doi:10.1023/A:1015678432355
- Niks, RE. et al. Quantitative resistance to biotrophic filamentous plant pathogens: concepts, misconceptions, and mechanisms. Annu. Rev. Phytopathol (2015). doi:10.1146/annurev-phyto-080614-115928
- Schulthess, AW. et al. Genomics-informed prebreeding unlocks the diversity in genebanks for wheat improvement. Nat Genet, (2022). doi: 10.1038/s41588-022-01189-7.
- Rubiales, D., Niks R. E. Characterization of Lr34, a major gene conferring nonhypersensitive resistance to wheat leaf rust. Plant Dis., (1995). doi: 10.1094/PD-79-1208
- Torp, J. et al. Powdery mildew resistance genes in 106 Northwest European spring barley varieties. Royal Veterinary and Agricultural University Yearbook, *pp.* 75–102. Copenhagen, Denmark. (1978)

Revised Manuscript (Track changes)
Click here to access/download;Personal Cover;GB2.0_Data-Publ_Giga_revised_TC.docx

Title

High throughput imaging of powdery mildew resistance of the winter wheat collection hosted at the *German Federal ex situ Genebank for Agricultural and Horticultural Crops*~~Powdery mildew resistance phenotyping of the winter wheat collection hosted at the German *Federal ex situ Genebank for Agricultural and Horticultural Crops* at IPK Gatersleben~~

Formatted: Font: Italic

Authors

Valentin Hinterberger[1](hinterberger@ipk-gatersleben.de),
Dimitar Douchkov[1] (douchkov@ipk-gatersleben.de),
Stefanie Lueck[1] (lueck@ipk-gatersleben.de),
Jochen C. Reif[1] (reif@ipk-gatersleben.de),
and Albert W. Schulthess[1,*] (schulthess@ipk-gatersleben.de)

Affiliations
[1] *Leibniz Institute of Plant Genetics and Crop Plant Research (IPK), D-06466, Seeland, Germany*
corresponding author: Albert W. Schulthess (schulthess@ipk-gatersleben.de)

Formatted: Font: 11 pt

Abstract

Genebanks worldwide are transforming into bio-digital resource centres, providing not only access to the plant material itself but also to its phenotypic and genotypic information. Adding information for relevant traits will help boosting plant genetic resources' usage in breeding and research.
Resistance traits are vital for adapting our agricultural systems to future challenges.
Here we provide phenotypic data for the resistance against *Blumeria graminis,* the causal agent of powdery mildew - a substantial risk to our agricultural production.
Using a modern ~~high-~~high-throughput phenotyping system, we infected and photographed a total of 113,638 wheat leaves of 7,505 winter wheat (*Triticum aestivum* L.) plant genetic resources of the *German Federal Ex Situ Geneb ~~B~~ank for Agricultural and Horticultural Crops~~, Germany~~* and 154 commercial genotypes. We quantified the resistance reaction captured by images and provide them here, along with the raw ~~pictures~~images.

Formatted: Left

This massive amount of phenotypic data combined with ~~the~~ already published genotypic data also provides a valuable and unique training dataset for the development of novel genotype-based predictions as well as mapping methods.

Background

Our agricultural system is facing one of the most significant upheavals in decades. In addition to uncertainties arising from ongoing climatic change and the ever-increasing demand for agricultural goods, the ecological impact of agricultural production is more than ever in the spotlight. In this context, ~~The~~ the European "Farm to Fork Strategy" has set ambitious goals for a more sustainable agricultural production. One of these goals is to reduce pesticide use by 50% by 2030 (EU commission, 2020).

Formatted: Font: 11 pt

Fungicides form an important group of pesticides in cereal crops, which have been used regularly in intensive agriculture since the mid-1970s. The reasons why there is an urgent need to reduce the use of fungicides are manifold: harmful pesticide residues (Cabrera ~~&~~ and Pastor, 2022), decreasing efficacy of active components due to pathogenic resistance (Lucas et al., 2015), and side effects on the environment and the crop (Calonne et al., 2011, Ullah et al., 2019) are just some of them.

There are many agronomical ways to reduce fungicide usage, e.g. precision farming (Zanin et al., 2022), improved crop rotation, changes in sowing date, and straw management. Growing resistant varieties is one of the easiest and most sustainable solutions for the farmer.

While easy to adopt for the farmer, breeding a stable resistant variety with excellent quality and high yield is a great challenge for breeders and phytopathologists.

The past decades have shown continuous cycles of a "Boom and Bust" pattern in resistance development - new major qualitative resistance mechanisms are identified and heavily used in agriculture. This has led to a strong selection pressure on the pathogen population and an inevitable break down of the resistance by population shift and mutations (McDonald and Linde, 2002, Wolfe, 1984). Especially b~~B~~iotrophic pathogens like *Blumeria graminis,* the causal agent of powdery mildew (PM), show a rapid and strong response to deploying of new resistance mechanisms (Wolfe, 1984). In this context, ~~The~~ the risk of pathogen populations adapting to resistance mechanisms ~~correlates negatively with~~can be delayed by ~~the~~ increasing diversity of the resistance mechanisms in cultivars and relying on ~~used~~ quantitative resistance provided by the additive effect of several minor resistance genes (Lucas et al., 2015, ~~). Therefore, increasing resistance diversity is one way to stabilize yields without fungicides.~~

McDonald and Linde 2002).

P~~Providing~~ roviding donors for new, unused, or since a long-time abandoned resistance genes is one of the main purposes of genebanks like the *German Federal* ~~*ex*~~ *Ex situ* ~~*g*~~*Gene* ~~-~~*bank for Agricultural and Horticultural Crops*~~, *Germany*~~. ~~However,~~The great challenge for breeders and scientists lies here on finding useful plant genetic resources (PGR) ~~in~~ among thousands of genebank accessions ~~provided by genebanks is a challenge for breeders and scientists~~. ~~On the one side,~~

~~Since the detached leaf assays are a standard method in phytopathology to assess plant resistance in a cheap, fast, easy, and repeatable manner (Torp et al., 1978). Detached leaf assays are traditionally performed to measure the qualitative resistance response at the seedling stage of plants. a standard method in phytopathology, cheap, easy, and repeatable quantification methods are highly desirable.On the other hand, there is evidence for quantitative resistance mechanisms in seedlings. For example, *Lr34* confers partial resistance already at the seedling stage (Rubiales and Niks, 1995) while some *SWEET* genes have been associated to quantitative susceptibility in seedlings (Chen et al., 2014, Gupta, 2020). Some of those quantitative or partial resistance mechanisms have a delaying (latency) effect on the development of the pathogen, resulting in longer reproduction cycles and a reduced spore production by the pathogen (Niks et al., 2015). We therefore investigated the plausibility of capturing latency mechanisms of quantitative resistance against PM at the seedling stage in a detached leaf assay setup applied at a large-~~

~~scale to genebank material.~~~~Therefore~~~~Here~~In order to make these informed prebreeding decisions possible, we have tested almost all of IPK's winter wheat~~, we tested almost the entire IPK winter wheat~~ (*Triticum aestivum* L.) collection for its quantitative resistance to PM by combining high-throughput imaging of detached leaf assays ~~experiments~~ and a machine-based quantification of the percentage of infected leaf area.

In this process, we infected and photographed a total of 113,638 wheat leaves of 7,505 accessions and 154 varieties used by farmers in Germany ~~in~~ during the last decades. This data was obtained in a controlled environment at the seedling stage and ~~tested against~~using the highly ~~aggressive~~ virulent PM isolate FAL 92315. Under this highly controlled setup and provided a strong genotypic effect of host plants, fungal growth could be attributed to a quantitative resistance response of genotypes.~~The defined virulence spectrum of this isolate provides advantages over field data that usually rely on natural infections and much less controlled environmental conditions.~~.

~~We performed the detached leaf assay using seedlings for this phenotyping (Torp et al. 1978). Usually this assay is used to phenotype qualitative resistance. We used this assay to phenotype quantitative resistance in seedlings.~~

~~Our hypothesis is that there are quantitative resistance mechanisms already present in seedlings, this is indicated by, for example, *Lr34* (Rubiales and Niks, 1995) which confers partial resistance already in seedling stage or some *sweet* genes which confer quantitative susceptibility (Chen et al., 2014 Gupta, 2020) and are also expressed in seedling stage. Some of those quantitative or partial resistance mechanisms have a delaying effect on the development of the pathogen resulting in a longer generation cycle in the field and a reduced spore production (Niks et al. 2015).~~

~~As we are using one defined isolate with known virulence spectrum in controlled conditions, we are hypothesizing that this latency effect should be part of our phenotype as less fungal matter should be present, as we are strictly controlling the developmental conditions.~~ Such a reliable association would most likely not be possible based on field data that rely on natural infections and much less controlled environmental conditions.

Detached leaf assays are a standard method in phytopathology to assess plant resistance in a cheap, fast, easy, and repeatable manner (Torp et al., 1978). They are traditionally performed to measure the qualitative resistance response at the seedling stage of plants. However, there is evidence for quantitative resistance mechanisms in seedlings. For example, *Lr34* confers partial resistance already at the seedling stage (Rubiales and Niks, 1995) while some *SWEET* genes have been associated to quantitative susceptibility in seedlings (Chen et al., 2014, Gupta, 2020). Some of those quantitative or partial resistance mechanisms have a delaying (latency) effect on the development of the pathogen, resulting in longer reproduction cycles and a reduced spore production by the pathogen (Niks et al., 2015). We therefore investigated the plausibility of capturing latency mechanisms of quantitative resistance against PM at the seedling stage in a detached leaf assay setup applied at a large-scale to genebank material.

~~The defined virulence spectrum of this isolate provides advantages over field data that usually rely on natural infections and much less controlled environmental conditions.~~ The here presented data can be further extended ~~for~~with additional untested plant material by ~~Furthermore, it is possible to reproduce this assay with additional lines in the future~~ using the same environmental parameters and isolate. In addition, this dataset may help to develop or train new image analysis tools for images derived from detached leaf assays. In combination with additional analysis using other isolates of *Blumeria graminis*, it can be part of a genotype-by-genotype analysis elucidating host-pathogen interactions.
~~The past decades has shown continuous cycles of a "Boom and Bust" pattern in resistance development - New major qualitative resistance mechanisms are identified and heavily used in agriculture. This has led to a strong selection preasure on the pathogen population and an inevitable break of the resistance by~~

Formatted: Font: Italic
Formatted: Not Highlight
Formatted: Not Highlight
Formatted: Font: Italic
Formatted: Font: Italic
Formatted: Not Highlight
Formatted: Not Highlight

~~population shift and mutations. Quantitative resistance consists of many minor resistance genes with very diverse mechanisms, which is considered more durable (McDonald and Linde 2002)~~ ~~We expect that our quantitative resistance data contribute to the future discovery of basal resistance mechanisms that provide a more durable protection in the future.~~

Formatted: Font: Calibri, 11 pt

As a component of genome-wide mapping approaches, this data is a valuable source of information on donors for potentially novel resistance genes, as we recently have shown (Hinterberger et al., 2022). We expect that our quantitative resistance data contribute to the ~~the future~~ discovery of basal resistance mechanisms that provide a more durable crop protection in the future.

~~In addition, this dataset may help to develop or train new image analysis tools for images derived from detached leaf assays. Since the detached leaf assays are a standard method in phytopathology, cheap, easy, and repeatable quantification methods are highly desirable.~~

Methods

Plant material

The *German Federal Ex Situ Genebank for Agricultural and Horticultural Crop Species*~~, Germany,~~ ~~hosted~~ located at the Leibniz Institute of Plant Genetics and Crop Plant Research (IPK)~~-~~ hosts more than 27,000 wheat PGR of the *Triticum* ~~*aestivum*~~ *sp.* ~~L. plant genetic resources (PGR~~genus~~)~~ (Sharma et al., 2021). In this study we present phenotypic data for powdery mildew resistance of 7,505 wheat PGR and 154 winter wheat varieties representing the cultivated varieties in Germany in the last decade (in the following denoted as the Elite Panel). In addition, a set of 929 additional genotypes (coded as Div_Set_1 ~~-~~– 929) were also tested in experiments but were not part of the study. Phenotypes of these additional genotypes were kept in the dataset to not disrupt the data structure and to allow proper correction for experimental design effects.

Formatted: Font: Italic

During field multiplication of genebank material, ~~As genbank accessions are sometimes mixtures of different genotypes rather than genetic homogeneous lines, w~~w ~~W~~e used a ~~"~~“single seed descent~~"~~” (SSD) step to ~~reduce genetic heterogeneity~~obtain defined seeds (for details, see Schulthess et al., 2022). This was achieved by bagging one representative ear ~~within~~for each of 7,502~~3~~ homogenous accessions and two ears in case of only three ~~by sampling one genotype per homogenous accessions and two genotypes if~~ accessions, which we identified as clearly heterogenous based on the morphological appearance of plants within each accession~~ were morphologically heterogenous (This was only done in three cases). In this step, we multiplied the seed samples in the field and selected one representative ear for further propagation (for details, see Schulthess et al., 2022~~). ~~Defined~~These defined seeds were also used for genotyping-by-sequencing (GBS) in a companion study (Schulthess et al., 2022). ~~The genetically uniform PGR were then used for phenotyping and genotyping-by-sequencing (GBS).~~ For the genotypes of the Elite Panel, defined seeds were obtained from local seed market providers~~ therefore genetic homogeneity is assumed in this material~~.~~The genetically uniform PGR were then used for phenotyping and genotyping-by-sequencing (GBS).~~

High-throughput phenotyping of plant-pathogen interactions

The phenotypic data presented here was gathered using the Macrobot facility, a robotic platform performing high-throughput semi-automatic detached leaf assays (Lueck et al., 2020a,b). For the Macrobot assay, seedlings from defined seeds were grown in~~ a~~ trays with 6 × 4 slots in the greenhouse under standardized conditions. In each slot ten seedlings of the same genotype were grown. For the inoculation assay, a leaf segment~~ves~~ was~~ere~~ cut from the second leaf of the 14-day-old seedlings. We cut the middle part of the leaf~~,~~ because early trials evidenced that the base of the leaf is more susceptible to powdery mildew, while the tip is more resistant (data not shown). ~~From those leaves t~~The two-cm-long

Formatted: Font: (Default) +Body (Calibri)

Formatted: Font: (Default) +Body (Calibri), 11 pt

leaf segments were brought onto microtiter agar plates. Each plate consisted of four lanes, each with leaf segments from up to eight leaves per tested genotype. These plates were then infected with highly virulent *Blumeria graminis f. sp. tritici* isolate FAL 92315 (please see Supplementary Table 1 for the respective virulence/avirulence spectrum) in a rotating platform by blowing spores from heavily infected leaves using a compressed air pistol.

The maximum capacity of the inoculation tower of twelve plates defines the size of an independent experiment. Since each tray corresponds to six plates, two trays formed an independent experiment (see ~~figure~~ Figure 1 for a graphical illustration). The inoculated plates were incubated for six days in an incubation chamber under ~~controlled~~ standardized conditions (20°C, 60% RH, 16 h photoperiod, 15 $\mu E\ m^{-2}\ s^{-1}$). After this incubation time, images (3296 × 2472 pixel) were acquired using an RGB-Camera and stored in 16-bit TIFF format (details of the used hardware are described in Lueck et al., 2020b).

Based on the image data, the percentage of infected leaf area was determined by developing an open-source algorithm ~~trained and~~ implemented in Python (Lueck et al., 2020a).

The independent experiments were linked by the susceptible cultivar KANZLER, which was also used for quality control. KANZLER was tested four times in each 24-slot tray, i.e. eight times per experiment. In addition, to increase the reliability of the generated phenotypic data obtained, each genotype was tested in two or more independent experiments.

Data curation of phenotypic data

To improve the quality of the data presented here, we developed and implemented an automatic stepwise quality control in the R environment (R Core Team, 2020)~~. This script is also available within the data repository~~. First, we double-checked that the data structure and data format present in the recorded measurements and metadata correspond with the actual design of phenotyping experiments. At this step, we controlled if lanes had a minimum number of three leaves and plates contained an exact number of four lanes. We also checked for errors in the label or lane detection of the automatic picture analysis and manual errors in the metadata.

Data points that met these criteria were tested afterwards for ~~their quality~~the presence of outliers ~~in~~ at three different levels~~three~~ (steps)~~.~~:

In the first step, we tested the distribution of technical replicates of a measurement (up to eight leaves per lane). We excluded outliers by using 1.5 times the interquartile distance as a threshold.

In the second step, we evaluated the data quality at the experiment level. There, we excluded whole experiments based on the infection of the susceptible control genotype KANZLER. The rationale behind this was, that if the infection level of KANZLER is low, the inoculation of the experiment failed. To ~~define~~ detect outliers here, we defined a threshold for the mean and maximal values of the control of each experiment by using the 1.5 interquartile distance or the infected leaf area again.

The third and final quality control step was based on the variance between the biological replicates (so the same genotype was tested in two different experiments). To do so, we fitted the same model as for best linear unbiased estimation (BLUEs) and variance component estimation (see Equation 1) and defined a significant outlier threshold (p-value < 0.01) for the residuals of fitted genotypic means based on Anscombe and Tukey (1963).

All computational methods were performed within the R environment (R Core Team, 2020 version 4.0.2. using R-Studio version 1.3.1056).

Formatted: Not Highlight

Formatted: Indent: Left: 0", Right: 0", Line spacing: single

Best linear unbiased estimation and variance components estimation

To estimate the effect of the design parameters and correct the phenotypic values for those, we estimated the variance components and the BLUEs of the genotypes using the phenotypic data. BLUEs of the genotypes and variance components were estimated based on the curated data. For the estimation of variance components of the percentage of infected leaf area, we used the following linear mixed model (Hinterberger et al., 2022):

~~(1)~~

$$y = \mu + genotype + experiment + tray(experiment) + error, \qquad (1)$$

where the common mean (μ) was treated as a fixed factor, whereas genotype, experiment, the tray nested within an experiment, and error effect were assumed as random factors. BLUEs were computed using the same model but assuming the genotype factor as a fixed effect. All linear mixed models were solved using the ASReml-R package Version 4 (Butler et al., 2017).
The heritability was estimated as in the following equation:

~~(2)~~

$$h^2 = \frac{\sigma_G^2}{\sigma_G^2 + \frac{\sigma_e^2}{R}} \qquad (2)$$

where $\sigma_G{}^2$ is the genotypic variance, $\sigma_e{}^2$ is the residual variance while R represents the average number of replications (independent experiments) per genotype. The standard deviation of the heritability was estimated using a bootstrapping approach by performing 500 heritability estimations using random samples that contained 80% of the total number of genotypes.

Genomic-phenomic data interoperability

In addition to the heritability as an indicator of data quality, we also assessed the genomic-phenomic data interoperability based on the genomic best linear unbiased prediction (GBLUP) for leaf infections ~~using a bootstrapping approach.~~ ~~We~~ and ~~used~~ using ~~the~~ ~~publically~~publicly available ~~(Schulthess et al., 20221) single nucleotide polymorphisms (SNPs) data generated in a genotyping-by-sequencing approach~~GBS data (Schulthess et al., 2022).
For this prediction, we used a GBLUP model implemented in the kin.blup()-function, a wrapper for the mixed.solve()-function in the rrBLUP-Package (Endelman, 2011). The fitted mixed model can be described as follows:

~~(3)~~

$$Y = \mathbf{1}_n\mu + \boldsymbol{Z}g + e \qquad (3)$$

~~Where~~ where Y stands for a vector of trait values for n genotypes, $\mathbf{1}_n$ is a unit vector, μ ~~stands~~ corresponds ~~for~~ to the population mean, $\boldsymbol{Z}$ ~~stands~~ indicates ~~for~~ a design matrix linking the elements of g to Y, g ($g \sim N(0, \sigma_g^2\mathbf{G})$) ~~stands for~~is a vector of random genotypic values and e ($e \sim N(0, \sigma_e^2\boldsymbol{I})$) ~~is~~ accounts for the

Formatted ...
Formatted: Font: (Default) +Body (Calibri), 11 pt
Formatted: Font: 11 pt
Formatted: Right
Formatted ...
Formatted: Font: (Default) +Body (Calibri)
Formatted: Font: (Default) +Body (Calibri)
Formatted: Right
Formatted: Font: (Default) +Body (Calibri), 11 pt
Formatted ...
Formatted ...
Formatted: Font: (Default) +Body (Calibri), 11 pt
Formatted: Right, Indent: Left: 0"
Formatted: Font: (Default) +Body (Calibri)
Formatted: Font: (Default) +Body (Calibri)
Formatted: Font: (Default) +Body (Calibri)
Formatted: Font: 11 pt
Formatted: Justified
Formatted: Font: (Default) +Body (Calibri)
Formatted: Font: 11 pt
Formatted: Right
Formatted ...
Formatted: Font: (Default) +Body (Calibri)
Formatted: Justified
Formatted: Font: (Default) +Body (Calibri)
Formatted: Font: (Default) +Body (Calibri)
Formatted: Font: (Default) +Body (Calibri)
Formatted: Font: (Default) +Body (Calibri)
Formatted ...

random residual term. **G** represents an additive genomic relationship matrix~~,~~ based on GBS marker and calculated ~~(coded as -1,0,1), here~~according to the first method of VanRaden (VanRaden, 2008)~~ was used~~. ***I*** stands for an identity matrix, while σ_g^2 and σ_e^2 are the genotypic and error variance components, respectively.

The assessment of the genomic-phenomic data interoperability ~~prediction~~ was performed ~~as~~using a 5-~~-~~fold cross validation approach. The “fold” means in how many subparts we split the dataset: in our case, the dataset was randomly split into five parts in each cross-validation run. In more detail, the genomic and phenotypic data of the first four parts were used as training set to predict the (fifth) remaining part (called test set) based only on the genomic data. Predictions were then compared with the observed phenotypes of the test set through correlation. The assignment of four parts to the training set and the fifth part to the test set was permutated in such a way that each subdivision served as test set only once and was four times part of the training set. The mean correlation between predicted and observed values from the five different permutations ~~The prediction was performed by randomly splitting the dataset into 5 parts of similar size. Each of those parts were than predicted using the other 4 parts of the dataset to train the model. As accuracy measurement we used the correlation of the testset prediction (the predicted fifth of the dataset) prediction and the measured values. Out of those 5 predictions the mean value~~ was saved for each run. We performed 500 runs of this procedure.

Data description

The here described raw data as well as BLUEs, the raw ~~pictures~~ images from the detached leaf assay, and the R-~~S~~ script to import and curate the raw phenotypic data ~~is~~ are available in the e!DAL-PGP-Repository (Arend et al., 2014) and can be directly accessed here (https://doi.ipk-gatersleben.de/DOI/be08fbd6-4885-4f19-a849-aac73915619b/8fe440c8-6e2d-490c-841f-122bef47dfc1/2/1847940088). In more detail, ~~*(Note: This is not the final DOI, it will be generated as soon as the review process is completed)*~~

~~T~~the repository contains the raw images of the individual measured leaves, the raw values of the predicted infected leaf area by the open-source Python implementation of Lueck et al., 2020a, and the curated, ready-to-use data in the form of BLUEs. We also provide the images of the whole plates.

To comply with the FAIR principles, the data were described according to the ISA-Tab format (Sansone et al., 2012).

This includes an investigation file ("i_investigation.txt") with general information about the conditions under which the data was produced ~~ere created~~ and a description of the protocols used to generate and curate the presented data. The experimental conditions and design effects of the ~~high~~ high-throughput assay are described in the corresponding study file ("s_GB2.0_MACRO_PM.txt"). The corresponding genotype identifiers to the previously published genotypic data for the population (Schulthess et al., 202~~1~~2) are also provided here. The assay file ("a_GB2.0_MACRO_PM.txt") contains the predicted infected leaf area and the corresponding image identifier for each leaf value. In addition to that, we added the minimal, mean and maximal average daily temperatures during the greenhouse period of each tested genotype to the data.

Specifically, the study file includes the effects of the experimental design of the ~~Macrobot~~ Macrobot assay, namely the Experiments ID, the Tray ID, and the Replication Nr. Besides these, we provide the sowing, inoculation and measuring dates. The "Source Name" is the ~~Accession~~ accession number from the IPK Genebank Documentation System (GBIS) combined with an internal project number reflecting the defined seed (~~derived~~ SSD in case of PGR)~~line from the accession~~. ~~We also added the GBIS DOI, a direct link to more information about the accession from the Genebank Information System at the IPK.~~ ~~As~~Detecting mislabeling, duplicates, and correcting ~~mislabeling~~ passport data is a well-~~-~~known ~~problem~~challenge ~~in~~for genebanks worldwide (Schulthess et al. 2022)~~,~~. For example, ~~C~~changes of ~~for example~~ the origin

Formatted: English (United States)

Commented [AS1]: Note: This is not the final DOI. The final DOI will be generated as soon as the review process is completed.

Formatted: Not Highlight

Formatted: Highlight

Formatted: Highlight

Formatted: Adjust space between Latin and Asian text, Adjust space between Asian text and numbers

Formatted: Font: Not Italic

information or genotype names happen regularly. GBIS is therefore a constantly curated system and works with unique . digital object identifiers (DOI) to exactly trace back requested plant material to the source accessions and their information. WChanges of for example the origin information or genotype names happen regularly. Therefore we include GBIS DOIs as part of the data and encourage readers and users to use the GBIS DOIm and not theinstead of genotype names to get further information and request on interesting genotypesPGR for further research and breeding activities. This link also allows direct ordering of accessions of interest for scientific and breeding purposes. In addition, SAMEA (SAM, BioSample accession; E, EBI; A, Assay Sample) numbers that link phenotypes to raw sequence reads are included. Sequence data can be accessed through SAMEA numbers at https://www.ebi.ac.uk/biosamples/.
The "Sample Name" is a unique identifier, connecting the genotype ID in the study-file with the raw phenotypic values in the assay file. It is also the name of the corresponding raw image.
In addition, we also provide the phenotypic data in a .csv file "raw_phenotype.csv", which is used as input by the provided R-Script.
We also give access to the BLUEs for the percentage of infected leaf area based on the curated raw data. These estimates are ready-to-use for different purposes (e.g., resistance donor selection, mapping approaches, of resistance donor selectionor genomic prediction).

Image data

The images generated by the Macrobot facility are the starting point for the analyses conducted. They were acquired using a Thorlabs 8050M-GE-TE camera at a resolution of 3,296 × 2,472 pixels with 365 nm (UV), 470 nm (blue), 530 nm (green), and 625 nm (red) peak wavelengths, and white light back illumination (for more details, see Lueck et al., 2020b).
The raw pictures of the whole plates are saved in 16-bit TIFF format and are provided in the same repository. . We We separatedcut out the images of individual leaf ves positions from full plate images to allow a datapoint-wise connection of phenotypic data (percentage of infected leaf area) and picture data. Those images are also provided here in PNG-format. Both sets of images have an expected re image resolution of 25 pixels/mm. .
The infected leaf area was predicteddetermined on those images using the image analysis pipeline described in Lueck et al. (2020a).

Phenotypic data

The phenotypic data presented here concern the quantification of the infected leaf area. These data show the quantitative host-pathogen interaction in a controlled environment. Raw values range from 0 to 98 % infected leaf area with a mean for the whole dataset of 48.16 % (Figure 2 and 3). We observed a lower mean for the tested Elite Panel (31.87 %) and a slightly lower maximum value (94 %). In total, we measured 113,638 leaves in 422 independent experiments (Table 1) connected through the control genotype KANZLER. On average, each genotype was tested in 1.95 experiments, with seven genotypes some tested up to 6 six times and others 418 tested only once. That a genotype, besides KANZLER, In the case of the over tested genotypes, this was unexpectedly tested in more than two independent experiments was due to few imparities due to imparities during seed logistics that could not be early detected because the handled material was coded. . In the case of the only once tested genotypes tested in no more than one experiment, this was mostly due to seed availability and/or germination issues. After outlier correction, 93.4% of the raw data were considered reliable and therefore used to compute BLUEs. We excluded 3,013 datapoints (measurements of leaves) (2.7%) due to outlier correction performed based on the technical replications. Due to failed experiments, we excluded 3,827 datapoints, ori.e. 3.3% of the total data collected, while . And due to high differences between the biological replications we excluded 630

datapoints (0.6% of the total data) were excluded due to high differences between the biological replications.

Technical validation

We used two criteria to evaluate the data quality presented here: firstfirst, heritability, and second, cross-validated genomic prediction.

-The achieved heritability of the measured host-pathogen interaction was 0.75. Variance components analysis revealed a high effect of the experimental design on the raw phenotypes. (Table 1). The performed data curation decreased the magnitude of the "“Experiment"” and residual effects increased in turn the variation proportion explained by the "“Genotype"” effect. -This high heritability and the Gaussian-like distribution of the genotypic means or BLUEs (Figure 3) supports the quantitative nature of the resistance response against PM already at seedling stage.

To evaluate the Ggenomic-phenomic data interoperabilitygenomic-phenomic- data-interoperability, we performed 500 runs of cross-validated genomic prediction. This analysis revealed a high prediction accuracy after data curation 0.507±0.004. In this regard, a 0.4% boost in accuracy could be attributed to the data curation steps.

Summary and outlook

We provide quantitative resistance phenotypes for 7,505 accessions of winter wheat against *Blumeria graminis*, causing PM infection at the seedling stage. Moreover, Quantitative resistance is often considered to play only a role in adult plants, while seedlings only show qualitative resistance (Niks et al. 2015). Wwe showed, that thise quantification of PM resistance in seedlings using is possible and reliable using detached leaf assays – an approach traditionally used to characterize qualitative resistance is possible. and the performed data quality assessments grant its reliable datality. This large dataset of a diverse host population tested with one Blumeria Graminis isolate grants highly repeatable data showing the existence of quantitative resistance response already at seedling stage. This is indicated also by the gausian like distribution of the phenotype data (Figure 3). ButHowever, we also seethe method has also some of the -limitations of- detached leaf assays in seedlings in general:

- i) the method and of the dataset presented here. First of all, this method is best It is bestmostly well-suited tofor foliar diseases like leaf rust and stem rust, besides powdery mildew..
- -
- The weak to moderate correlation between our high-throughput data - obtained under artificial controlled conditions with a single isolate - and field data – fully relying on natural infections (Hinterberger et al. 2022) indicates that our data should not be directly interpreted as field resistance. This most likely because natural infections are the result of a diverse population of multiple pathotypes interacting with a changing environment and the crop. We therefore presume that Also, the here gathered phenotypes are not a direct proxy for field resistance or adult plant resistance. This is due to two main reasons.
- First, although the isolate is highly virulent (see supplementary table 1), it does not show host genotype response to a diverse pathogen population. There is not enough information available to estimate how the global pathogen population is consisted, so it remains unclear how relevant the used isolate is in praxis.
- While we will focus more on the topic of resistance breeding and research in the future and tackle the problem using open data, we will be able to testing different individual isolates, which are dominant in the current pathogen gene pool,- could contribute to reduce this limitationusing the here presented approach.

Formatted: Font: Italic

Formatted: Bulleted + Level: 1 + Aligned at: 0.25" + Indent at: 0.5"

The assessed quantitative resistance could provide crop plant protection effects by delaying the development of the pathogen population~~s~~. ~~Secondly, there are of cause differences in the resistance response of seedlings compared with adult plants.~~

~~Never the less~~All in all, the here presented dataset, in combination with already available gen~~etic~~omic information and the possibility to connect the results from this assay with other studies using the PGR population of the IPK, will serve as a good base for an educated ~~guesses to select new mapping populations~~selection. Considering the diverse origins of the phenotyped plant genetic resources (Schulthess et al. 2022) we ~~furthermore hope to~~expect to provide a valuable resource for breeders and scientists in different global regions.

Code availability

All computational methods were performed within R environment (R Core Team, 2020, version 4.0.2~~.~~ using R-Studio version 1.3.1056). The c~~C~~ode to import and curate the data (“GB2.0_Macro_PM_15.06.2022.R”) is also available ~~in~~ at ~~the same~~ e!DAL~~-repository~~ (https://doi.ipk-gatersleben.de/DOI/dc5316a5-aad7-423b-9ce7-2d972acc0ac8/182f2ae0-6879-4f9a-980a-2c217c8e8c6b/2/1847940088). ~~*(Note: This is not the final DOI, it will be generated as soon as the review process is completed)*~~

Field Code Changed

Commented [AS2]: Note: This is not the final DOI. The final DOI will be generated as soon as the review process is completed.

Formatted: Right: 0", Line spacing: single

Acknowledgements

The experimental work was supported by the German Federal Ministry of Education and Research within the GeneBank2.0 Project (Grant Nos. FKZ031B0184B and FKZ031B0184A) and supported by the German Plant Phenotyping Network (DPPN) (FKZ 031A053).

We thank Daniel Arend for his bioinformatic support and would like to acknowledge the following colleagues for the valuable technical help ~~of in~~during the course of performing ~~the~~ of experiments (in alphabetical order): Md. Al Mamum, Sonja Alner, Evangeline G. Avogadro, Federico Barbier, Ruben Betz, Gabriele Brantin, Bettina Brückner, Alessia De Matteis, Deniz Demirhan, Birgit Dubsky, André Fessel, Lena Gaczensky, Christin-Sophie Gäde, Armand Garcia, Sonja Gentz, Kathrin Gramel-Koch, Bettina Kersten, Andrea Kunze , Martina Kühne, Gabriele Lange, Ingrid Marscheider, Liana Münchhoff, Jelena Perovic, Linda Ries, Gabriele Stentzel, Julia Sturz, Jacqueline Templer, Claudia Voigt and Ellen Weiss.

We also thank Moritz Lell for his bioinformatic support and the many fruitful discussions.

Author contributions

AWS and JCR designed the study; DD generated phenotypic data; SL performed the image analysis, VH curated the data, performed ~~the~~ quantitative genetic analyses, and wrote the manuscript with the input of all other authors.

Competing interests

The authors declare no conflict of interest.

~~Figures~~*Figure captions*

Figure 1: Schematic representation of the experimental design and the workflow of the Macrobot ~~high~~ high-throughput powdery mildew phenotyping (modified from Hinterberger et al., 2022)

Figure 2: Distribution of the raw and curated data that supports the exclusion of extreme/unexpected datapoints at levels: (1): Outlier(s) based on the technical replications of single genotypes; (2) Outlier experiment(s) based on the infection level of the susceptible control genotype; (3) Outlier(s) based on the difference in infection levels of the biological replications of single genotypes. The numbers at the top of the graph indicate the number of datapoints in each category (~~For~~ for details, see chapter: 'Data ~~Curation~~ curation of phenotypic data')

Figure 3: *~~Distribution~~Histogram of the best linear unbiased estimations of the percentage of infected leaf area of 7,505 ~~P~~plant genetic resources ~~presented here~~. The red dotted line represents the mean of the distribution (modified from Hinterberger et al., 2022)~~presented data.~~*

Formatted: Font: (Default) +Body (Calibri), 11 pt, Bold, Not Italic, Font color: Auto

Formatted: Font: (Default) +Body (Calibri), 11 pt, Not Italic, Font color: Auto

Formatted: Font: (Default) +Body (Calibri), 11 pt, Not Italic, Font color: Auto

Formatted: Font: (Default) +Body (Calibri), 11 pt, Not Italic, Font color: Auto

Formatted: Font: (Default) +Body (Calibri), 11 pt, Not Italic, Font color: Auto

Formatted: Font: (Default) +Body (Calibri), Bold

Formatted: Font: (Default) +Body (Calibri)

Formatted: Font: (Default) +Body (Calibri)

Formatted: Font: (Default) +Body (Calibri)

Formatted: Font: (Default) +Body (Calibri)

Formatted: Caption

Formatted: Font: (Default) +Body (Calibri)

Formatted: Font: (Default) +Body (Calibri), 11 pt, Not Bold, Font color: Auto

Table~~s~~

Table 1: Variance components and Heritability of the raw and curated phenotypic data. The factor "Experiment" refers to 446 independent experiments in which the data was generated. The Factor "Tray" refers to the tray in which the plants were grown together

~~*Table 1: Variance components and Heritability of the raw and curated phenotypic data. The factor "Experiment" refers to 446 independent experiments in which the data was generated. The Factor "Tray" refers to the tray in which the plants were grown together*~~

	Raw Data		Curated Data	
**Component**	**Estimation**	**SE**	**Estimation**	**SE**
Experiment	198.89	14.52	157.58	11.99
Experiment:Tray	25.46	2.29	26.03	2.35
Genotype	159.77	3.73	172.69	3.92
Residual	140.16	1.89	131.26	1.83
Heritability	0.73		0.75	
SD	0.005		0.005	
Genotypes(PGR)	7,505		7,464	
Genotypes(Elite)	154		154	
Experiments	422		405	
Plates	4,887		4,694	
Lanes	14,830		14,177	
Leaves	113,638		105,647	

Formatted: Font: 11 pt, Bold, Font color: Auto

Formatted: Normal

Formatted: Font: 11 pt, Font color: Auto

Formatted: Font: 11 pt, Font color: Auto

Formatted Table

Formatted Table

References:

[1] European commission. A farm to fork strategy for a fair healthy and environmentally-friendly food system; 2020; CELEX:52020DC0381.

[2] Cabrera LC, Pastor PM. The 2020 European Union report on pesticide residues in food. EFSA Journal. 2022; doi: 10.2903/j.efsa.2022.7215

[3] Lucas JA, Hawkins JN, Fraaije BA. The evolution of fungicide resistance. Adv. Appl. Microbiol. 2015; doi: 10.1016/bs.aambs.2014.09.001

[4] Calonne M, Fontaine J, Debiane D, et al. Side effects of the sterol biosynthesis inhibitor fungicide, propiconazole, on a beneficial arbuscular mycorrhizal fungus. Commun Agric Appl Biol Sci. 2011; PMID:22702206

[5] Ullah MR, Dijkstra FA. Fungicide and bactericide effects on carbon and nitrogen cycling in soils: a meta-analysis. Soil Syst. 2019; doi:10.3390/soilsystems3020023

[6] Zanin ARA, Neves DC, Teodoro LPR, et al. Reduction of pesticide application via real-time precision spraying. Sci Rep. 2022; doi:10.1038/s41598-022-09607-w

[7] McDonald BA, Linde C. The population genetics of plant pathogens and breeding strategies for durable resistance. Euphytica. 2002; doi:10.1023/A:1015678432355

[8] Wolfe MS. Trying to understand and control powdery mildew. Plant Pathol. 1984; doi:10.1111/j.1365-3059.1984.tb02868.x

[9] Torp, J. et al. Powdery mildew resistance genes in 106 Northwest European spring barley varieties. Royal Veterinary and Agricultural University Yearbook, pp. 75–102. Copenhagen, Denmark. (1978)

[10] Rubiales D, Niks RE. Characterization of Lr34, a major gene conferring nonhypersensitive resistance to wheat leaf rust. Plant Dis. 1995; doi: 10.1094/PD-79-1208

[11] Chen L. SWEET sugar transporters for phloem transport and pathogen nutrition. New Phytol. 2014; doi:10.1111/nph.12445

[12] Gupta PK. SWEET genes for disease resistance in plants. Trends Genet. 2020; doi:10.1016/j.tig.2020.08.007

[13] Niks RE., Qi XQ, Marcel TC. Quantitative resistance to biotrophic filamentous plant pathogens: concepts, misconceptions, and mechanisms. Annu Rev Phytopathol. 2015; doi:10.1146/annurev-phyto-080614-115928

[14] Hinterberger V, Douchkov D, Lueck S, et al. Mining for new sources of resistance to powdery mildew in genetic resources of winter wheat. Front Plant Sci. 2022; doi:10.3389/fpls.2022.836723

Formatted: English (United Kingdom)

Formatted: Spanish (Chile)

[15] Sharma S, Schulthess AW, Bassi FM, et al. Introducing beneficial alleles from plant genetic resources into the wheat germplasm. Biology. 2021; doi:10.3390/biology10100982

[16] Schulthess AW, Kale SM, Liu F, et al. Genomics-informed prebreeding unlocks the diversity in genebanks for wheat improvement. Nat Genet. 2022; doi: 10.1038/s41588-022-01189-7

[17] Lueck S, Beukert U, Douchkov D. BluVision Macro - a software for automated powdery mildew and rust disease quantification on detached leaves. J Open Source Softw. 2020a; doi:10.21105/joss.02259

[18] Lueck S, Strickert M, Lorbeer M, et al. "Macrobot": an automated segmentation-based system for powdery mildew disease quantification. Plant Phenomics. 2020b; doi:10.34133/2020/5839856

[19] R Core Team. R: A language and environment for statistical computing. R Foundation for Statistical Computing, Vienna, Austria. 2020; URL: https://www.R-project.org/

[20] Anscombe FJ, Tukey JW. The examination and analysis of residuals. Technometrics. 1963; doi:10.2307/1266059

[21] Butler DG, Cullis BR, Gilmour AR, et al. ASReml-R reference manual version 4. VSN International Ltd, Hemel Hempstead, HP1 1ES, UK. 2017; URL: https://asreml.kb.vsni.co.uk/wp-content/uploads/sites/3/ASReml-R-Reference-Manual-4.pdf.

[22] Endelman JB. Ridge regression and other kernels for genomic selection with R package rrBLUP. Plant Genome. 2011; doi:10.3835/plantgenome2011.08.0024

[23] VanRaden PM. Efficient methods to compute genomic predictions. J Dairy Sci. 2008; doi:10.3168/jds.2007-0980.

[24] Arend D, Lange M, Chen J, et al. e!DAL - a framework to store, share and publish research data. BMC Bioinformatics. 2014; doi:10.1186/1471-2105-15-214

[25] Sansone SA, Rocca-Serra P, Field D, et al. Toward interoperable bioscience data. Nat Genet. 2012; doi:10.1038/ng.1054

Formatted: English (United States)

Formatted: Space Before: 12 pt, After: 12 pt
