# Supplementary material for: High-throughput imaging of powdery mildew resistance of the winter wheat collection hosted at the German Federal ex situ Genebank for Agricultural and Horticultural Crops
Source: Gigascience. 2023 Mar 3;12:giad007. doi: 10.1093/gigascience/giad007 (PMC9984986; doi:10.1093/gigascience/giad007)
Supplement: giad007_Response_to_Reviewer_Comments_Original_Submission [file giad007_response_to_reviewer_comments_original_submission.pdf]

Author's Response To Reviewer Comments

Close

Dear Hans,

You will find our Cover Letter and detailed answers to the comments of reviewers in the attached document file "Cover_letter.docx" labelled as "Cover letter and response to reviewers". We do not know if all reviewers have access to the original files or only to the PDF which is automatically generated by the editorial manager system. Therefore, we included two versions of our manuscript: in the main (first) file all changes performed during revision are "accepted", hence not be seen, but readability is improved. In the second file (included at the end of the PDF, after our answers to the reviewers), all changes can be seen for transparency. We hope that this can make the review process more efficient.

Best regards,
Albert W. Schulthess

Close
