# Supplementary material for: High-throughput imaging of powdery mildew resistance of the winter wheat collection hosted at the German Federal ex situ Genebank for Agricultural and Horticultural Crops
Source: Gigascience. 2023 Mar 3;12:giad007. doi: 10.1093/gigascience/giad007 (PMC9984986; doi:10.1093/gigascience/giad007)
Supplement: giad007_Response_to_Reviewer_Comments_Revision_1 [file giad007_response_to_reviewer_comments_revision_1.pdf]

Author's Response To Reviewer Comments

Close

Dear Hans,

you can find a cover letter (including our responses to the editorial remarks) and the current revised version of the manuscript (without and with track changes) in the submission.

Best regards,
Albert

Close
