# Supplementary material for: High-throughput imaging of powdery mildew resistance of the winter wheat collection hosted at the German Federal ex situ Genebank for Agricultural and Horticultural Crops
Source: Gigascience. 2023 Mar 3;12:giad007. doi: 10.1093/gigascience/giad007 (PMC9984986; doi:10.1093/gigascience/giad007)
Supplement: giad007_Response_to_Reviewer_Comments_Revision_2 [file giad007_response_to_reviewer_comments_revision_2.pdf]

Author's Response To Reviewer Comments

Close

Dear Hans,

You can find in the attachment the current revised version of our manuscript. We restructured the abstract following the standard of GigaScience and provided now references as index numbers in the main text. In the meanwhile, our companion FAIR data publication of genomic and field data is already available, so we also included it as part of the references. We double checked again all numbers and statistics presented in the manuscript as well as in the e!DAL repository and corrected for the last inaccuracies. These minor changes do not affect the main conclusions of our work and you can follow them in our manuscript through the 'track changes' functionality of Microsoft Word. e!DAL does not allow for updates (either for the description nor the data) after the DOI link has been generated. We provide therefore an updated DOI link in the references. We are very sorry for this last inconvenience as well as our delayed response.

Many thanks for your time and interest

Best regards,
Albert

Close
