# Supplementary material for: High-throughput imaging of powdery mildew resistance of the winter wheat collection hosted at the German Federal ex situ Genebank for Agricultural and Horticultural Crops
Source: Gigascience. 2023 Mar 3;12:giad007. doi: 10.1093/gigascience/giad007 (PMC9984986; doi:10.1093/gigascience/giad007)
Supplement: giad007_Reviewer_2_Report_Original_Submission — Feng Hui -- 8/16/2022 Reviewed [file giad007_reviewer_2_report_original_submission.pdf]

Reviewer Report

Title: High throughput imaging of powdery mildew resistance of the winter wheat collection hosted at the German Federal ex situ Genebank for Agricultural and Horticultural Crops

Version: Original Submission **Date:** 8/16/2022

Reviewer name: Feng Hui

Reviewer Comments to Author:

This paper addresses the problem of resistance traits and provides phenotypic data on resistance to powdery mildew, providing a dataset for the development of genotype-based prediction and mapping methods.1. The resolution of Fig1 and Fig2 is too low.2. The result and summary part should be added.3. The article does not cite studies similar to the topic for comparison. What are the advantages and limitations of this topic for previous studies?4. The weather conditions during planting should be added.5. How does the sampling area and sampling time affect the experimental results?6. The overall flow chart of experiment should be added.7. Line 286, "some tested up to 6 times and others tested only once". Why did this happen?8. Line 287, What are the criteria for outlier correction?9. What is the fold of the cross verification? What is the basis for grouping?10. How much phenotypic data were extracted in this paper?11. How to do the "Genomic-phenomic data interoperability"? What is the result? It is better to attach the operation process and video.12. Each part of the content has been published separately. What is the innovation of this paper?

Level of Interest

Please indicate how interesting you found the manuscript: Choose an item.

Quality of Written English

Please indicate the quality of language in the manuscript: Choose an item.

Declaration of Competing Interests

Please complete a declaration of competing interests, considering the following questions:

- Have you in the past five years received reimbursements, fees, funding, or salary from an organisation that may in any way gain or lose financially from the publication of this manuscript, either now or in the future?
- Do you hold any stocks or shares in an organisation that may in any way gain or lose financially from the publication of this manuscript, either now or in the future?
- Do you hold or are you currently applying for any patents relating to the content of the manuscript?

- Have you received reimbursements, fees, funding, or salary from an organization that holds or has applied for patents relating to the content of the manuscript?
- Do you have any other financial competing interests?
- Do you have any non-financial competing interests in relation to this paper?

If you can answer no to all of the above, write 'I declare that I have no competing interests' below. If your reply is yes to any, please give details below.

I declare that I have no competing interests

I agree to the open peer review policy of the journal. I understand that my name will be included on my report to the authors and, if the manuscript is accepted for publication, my named report including any attachments I upload will be posted on the website along with the authors' responses. I agree for my report to be made available under an Open Access Creative Commons CC-BY license (http://creativecommons.org/licenses/by/4.0/). I understand that any comments which I do not wish to be included in my named report can be included as confidential comments to the editors, which will not be published.

Choose an item.

To further support our reviewers, we have joined with Publons, where you can gain additional credit to further highlight your hard work (see: https://publons.com/journal/530/gigascience). On publication of this paper, your review will be automatically added to Publons, you can then choose whether or not to claim your Publons credit. I understand this statement.

Yes Choose an item.
