# Supplementary material for: High-throughput imaging of powdery mildew resistance of the winter wheat collection hosted at the German Federal ex situ Genebank for Agricultural and Horticultural Crops
Source: Gigascience. 2023 Mar 3;12:giad007. doi: 10.1093/gigascience/giad007 (PMC9984986; doi:10.1093/gigascience/giad007)
Supplement: giad007_Reviewer_3_Report_Original_Submission — Rohit Mago, Ph.D. -- 8/18/2022 Reviewed [file giad007_reviewer_3_report_original_submission.pdf]

Reviewer Report

Title: High throughput imaging of powdery mildew resistance of the winter wheat collection hosted at the German Federal ex situ Genebank for Agricultural and Horticultural Crops

Version: Original Submission **Date:** 8/18/2022

Reviewer name: Rohit Mago, Ph.D.

Reviewer Comments to Author:

The manuscript provides phenotypic data for the resistance against powdery mildew on leaves of 7398 winter wheat accessions held at German Federal Ex Situ Gene Bank for Agricultural and an additional 150 commercial cultivars. The authors used a high throughput system for phenotyping and provide over 110,000 pictures at high resolution of these genotypes infected with powdery mildew race FAL 92315. The data will be useful for other researchers and breeders to pick resistant germplasm in their breeding programs and the phenotypic data set can be used for AI based training for novel genotype-based predictions as well as mapping methodsThe manuscript is well written and warrants publication. However, there are a few questions that authors should address in the manuscript including limitations of the technique1. The authors used only a single virulent powdery mildew race in their experiments thus creating a bias. While the results will be useful for breeders to use the genotypes in Germany, they may not be useful for breeders in other locations due to prevalence of other pathotypes2. The high throughput phenotyping may not be suitable for diseases where it is not possible to perform detached leaf assays like powdery mildew

Level of Interest

Please indicate how interesting you found the manuscript: Choose an item.

Quality of Written English

Please indicate the quality of language in the manuscript: Choose an item.

Declaration of Competing Interests

Please complete a declaration of competing interests, considering the following questions:

- Have you in the past five years received reimbursements, fees, funding, or salary from an organisation that may in any way gain or lose financially from the publication of this manuscript, either now or in the future?
- Do you hold any stocks or shares in an organisation that may in any way gain or lose financially from the publication of this manuscript, either now or in the future?
- Do you hold or are you currently applying for any patents relating to the content of the manuscript?

- Have you received reimbursements, fees, funding, or salary from an organization that holds or has applied for patents relating to the content of the manuscript?
- Do you have any other financial competing interests?
- Do you have any non-financial competing interests in relation to this paper?

If you can answer no to all of the above, write 'I declare that I have no competing interests' below. If your reply is yes to any, please give details below.

I declare that I have no competing interests

I agree to the open peer review policy of the journal. I understand that my name will be included on my report to the authors and, if the manuscript is accepted for publication, my named report including any attachments I upload will be posted on the website along with the authors' responses. I agree for my report to be made available under an Open Access Creative Commons CC-BY license (http://creativecommons.org/licenses/by/4.0/). I understand that any comments which I do not wish to be included in my named report can be included as confidential comments to the editors, which will not be published.

Choose an item.

To further support our reviewers, we have joined with Publons, where you can gain additional credit to further highlight your hard work (see: https://publons.com/journal/530/gigascience). On publication of this paper, your review will be automatically added to Publons, you can then choose whether or not to claim your Publons credit. I understand this statement.

Yes Choose an item.
