# Supplementary material for: High-throughput imaging of powdery mildew resistance of the winter wheat collection hosted at the German Federal ex situ Genebank for Agricultural and Horticultural Crops
Source: Gigascience. 2023 Mar 3;12:giad007. doi: 10.1093/gigascience/giad007 (PMC9984986; doi:10.1093/gigascience/giad007)
Supplement: giad007_Reviewer_4_Report_Original_Submission — Chris Armit -- 8/30/2022 Reviewed [file giad007_reviewer_4_report_original_submission.pdf]

Reviewer Report

Title: High throughput imaging of powdery mildew resistance of the winter wheat collection hosted at the German Federal ex situ Genebank for Agricultural and Horticultural Crops

Version: Original Submission **Date:** 8/30/2022

Reviewer name: Chris Armit

Reviewer Comments to Author:

This Data Note describes a high throughput imaging approach for evaluating of powdery mildew resistance in wheat. I was invited to check the quality of the image data, and this review reports on the image data aspect of the manuscript.In the manuscript, the authors state the following:"The images generated by the Macrobot facility are the starting point for the analyses conducted. They were acquired using a Thorlabs 8050M-GE-TE camera at a resolution of 3,296 Ã…~ 2,472px with 365 nm (UV), 470 nm (blue), 530 nm (green), and 625 nm (red) peak wavelengths, and white light back illumination (for more details, see Lueck et al., 2020b). The raw pictures of the whole plates are saved in 16-bit TIFF format. We separated the images of individual leaves to allow a datapoint-wise connection of phenotypic data and picture data. Those images are provided here in PNG-format."From this description, I was anticipating a high-resolution, multi-spectral imaging dataset of sufficient quality for plant phenomic image analysis. However, on downloading the image data from e!dal, I have observed that the PNG image data is of low-resolution, and extremely grainy. To illustrate with one example, the PNG image [20191029_122731_exp56_P12-6_02_7.png] in directory [exp56_6] is only 179 x 58 pixels.In the manuscript, the authors further state the following:"The infected leaf area was predicted on those images using the image analysis pipeline described in Lueck et al. 2020a."This sentence is ambiguous and I am unsure as to whether the phenomic analysis was performed on the high-resolution TIFF images, or the low-resolution PNG images.Consequently, I have two questions for the authors relating to this manuscript and the accompanying dataset.Question 1Can the authors please confirm whether the image analysis outlined in the manuscript was performed on high-resolution TIFF images? Alternatively, was the image analysis performed on low-resolution PNG images?Question 2Will the original 16-bit TIFF images of whole plates be made publicly available in support of this study?

Level of Interest

Please indicate how interesting you found the manuscript: Choose an item.

Quality of Written English

Please indicate the quality of language in the manuscript: Choose an item.

Declaration of Competing Interests

Please complete a declaration of competing interests, considering the following questions:

- Have you in the past five years received reimbursements, fees, funding, or salary from an organisation that may in any way gain or lose financially from the publication of this manuscript, either now or in the future?
- Do you hold any stocks or shares in an organisation that may in any way gain or lose financially from the publication of this manuscript, either now or in the future?
- Do you hold or are you currently applying for any patents relating to the content of the manuscript?
- Have you received reimbursements, fees, funding, or salary from an organization that holds or has applied for patents relating to the content of the manuscript?
- Do you have any other financial competing interests?
- Do you have any non-financial competing interests in relation to this paper?

If you can answer no to all of the above, write 'I declare that I have no competing interests' below. If your reply is yes to any, please give details below.

I work for GigaScience where I perform checks on the quality of image data and the image analysis approach. My funding is not dependent on the outcome of this review, and I declare that I have no competing interests.

I agree to the open peer review policy of the journal. I understand that my name will be included on my report to the authors and, if the manuscript is accepted for publication, my named report including any attachments I upload will be posted on the website along with the authors' responses. I agree for my report to be made available under an Open Access Creative Commons CC-BY license (http://creativecommons.org/licenses/by/4.0/). I understand that any comments which I do not wish to be included in my named report can be included as confidential comments to the editors, which will not be published.

Choose an item.

To further support our reviewers, we have joined with Publons, where you can gain additional credit to further highlight your hard work (see: https://publons.com/journal/530/gigascience). On publication of this paper, your review will be automatically added to Publons, you can then choose whether or not to claim your Publons credit. I understand this statement.

Yes Choose an item.
